# HSF1 is a prognostic determinant and therapeutic target in intrahepatic cholangiocarcinoma

**DOI:** 10.1186/s13046-024-03177-7

**Published:** 2024-09-06

**Authors:** Antonio Cigliano, Isabella Gigante, Marina Serra, Gianpaolo Vidili, Maria M. Simile, Sara Steinmann, Francesco Urigo, Eleonora Cossu, Giovanni M. Pes, Maria P. Dore, Silvia Ribback, Egle P. Milia, Elena Pizzuto, Serena Mancarella, Li Che, Rosa M. Pascale, Gianluigi Giannelli, Matthias Evert, Xin Chen, Diego F. Calvisi

**Affiliations:** 1 Department of Medicine, Surgery, and Pharmacy, University of Sassari, via P. Manzella 4, Sassari, 07100 Italy; 2 Institute of Pathology, University of Regensburg, Regensburg, Germany; 3 National Institute of Gastroenterology, IRCCS “Saverio de Bellis”, Castellana Grotte, Italy; 4 Department of Biomedical Sciences, University of Cagliari, Cagliari, Italy; 5 Institute of Pathology, University of Greifswald, Greifswald, Germany; 6 Department of Bioengineering and Therapeutic Sciences, University of California, San Francisco, CA USA; 7 University of Hawaii Cancer Center, Honolulu, USA

**Keywords:** Intrahepatic cholangiocarcinoma, HSF1, Mouse models, KRIBB-11, Navitoclax

## Abstract

**Background:**

Intrahepatic cholangiocarcinoma (iCCA) is a lethal primary liver tumor characterized by clinical aggressiveness, poor prognosis, and scarce therapeutic possibilities. Therefore, new treatments are urgently needed to render this disease curable. Since cumulating evidence supports the oncogenic properties of the Heat Shock Factor 1 (HSF1) transcription factor in various cancer types, we investigated its pathogenetic and therapeutic relevance in iCCA.

**Methods:**

Levels of HSF1 were evaluated in a vast collection of iCCA specimens. The effects of HSF1 inactivation on iCCA development in vivo were investigated using three established oncogene-driven iCCA mouse models. In addition, the impact of HSF1 suppression on tumor cells and tumor stroma was assessed in iCCA cell lines, human iCCA cancer-associated fibroblasts (hCAFs), and patient-derived organoids.

**Results:**

Human preinvasive, invasive, and metastatic iCCAs displayed widespread HSF1 upregulation, which was associated with a dismal prognosis of the patients. In addition, hydrodynamic injection of a dominant-negative form of HSF1 (HSF1dn), which suppresses HSF1 activity, significantly delayed cholangiocarcinogenesis in AKT/NICD, AKT/YAP, and AKT/TAZ mice. In iCCA cell lines, iCCA hCAFs, and patient-derived organoids, administration of the HSF1 inhibitor KRIBB-11 significantly reduced proliferation and induced apoptosis. Cell death was profoundly augmented by concomitant administration of the Bcl-xL/Bcl2/Bcl-w inhibitor ABT-263. Furthermore, KRIBB-11 reduced mitochondrial bioenergetics and glycolysis of iCCA cells.

**Conclusions:**

The present data underscore the critical pathogenetic, prognostic, and therapeutic role of HSF1 in cholangiocarcinogenesis.

**Supplementary Information:**

The online version contains supplementary material available at 10.1186/s13046-024-03177-7.

## Background

Intrahepatic cholangiocarcinoma (iCCA) is the second most common primary liver tumor, occurring proximal to the second-order bile ducts. It is characterized by increasing incidence worldwide, biological aggressiveness, high mortality, and limited therapeutic options. Primary risk factors for iCCA include primary sclerosing cholangitis, fibro-polycystic liver disease, hepatitis B and C virus chronic infection, hepatobiliary flukes, hepatolithiasis, metabolic dysfunction-associated steatotic liver disease (MASLD), and metabolic syndrome. However, a specific etiologic factor remains obscure in most cases, especially in Western countries. Consequently, targeted surveillance in a population with predisposing conditions is not feasible [1–4].

The prognosis for iCCA is most often dismal due to its asymptomatic nature in the early stages of the disease, which precludes surgical approaches, and the intrinsic resistance to chemotherapeutic agents by the tumor cells. In addition, early vascular invasion and metastasis by iCCA further contribute to poor patient outcomes, with the five-year survival rate falling below 20% [1–4].

Current systemic treatments for unresectable iCCA include chemotherapy, immunotherapy, and targeted therapy [5, 6]. In particular, chemo-immunotherapy is becoming the first-line standard of care for inoperable iCCA, based on the recommendations stemming from the phase III TOPAZ-1 and KEYNOTE-966 trials [6–8]. These studies revealed improved survival of iCCA patients subjected to first-line chemotherapy associated with the immune checkpoint inhibitors, compared with gemcitabine/cisplatin chemotherapy alone [6–8]. Nonetheless, the benefits of these therapeutic approaches remain far from satisfactory. Thus, investigations are urgently needed to identify and characterize novel actionable targets for iCCA treatment.

Heat Shock Factor 1 (HSF1) is a stress response transcription factor possessing oncogenic properties in various solid tumors, including breast, colon, lung, skin, liver, pancreas, myeloma, and prostate [9–11]. Due to HSF1’s ability to promote cell survival in stressful conditions, such as those faced by cancer cells during their growth, it is not surprising that HSF1 suppression is highly detrimental to tumor cells [9–11]. Furthermore, besides its role in the stress response, HSF1 activates a plethora of downstream effectors involved in cell proliferation, survival, and metabolism. For instance, HSF1 activates critical protooncogenes, such as c-Myc and FoxM1, which regulate multiple aspects of cell biology [9–13]. In addition, HSF1 controls many energy metabolism genes to promote glucose and lactate uptake. Also, HSF1 drives the preferential production of lactate, a phenomenon known as the Warburg effect. Therefore, activation of HSF1 can be considered a “cancer trait” and a biomarker of tumor aggressiveness [9–11].

In liver cancer, HSF1 upregulation has been found to correlate with tumor invasion and metastasis, as well as with patients’ prognosis, in hepatocellular carcinoma (HCC) [14–16]. Similarly, HSF1 is positively associated with the biological aggressiveness of pancreatic-biliary tumors and predicts a dismal outcome for iCCA patients [17, 18]. Furthermore, suppression of HSF1 inhibited hepatocellular carcinogenesis driven by c-Myc and AKT protooncogenes in the mouse [19, 20]. In the present study, we took a comprehensive approach to delineate the relevance of HSF1 in cholangiocarcinogenesis using human tumor specimens, mouse models, and in vitro systems.

Overall, the data indicate that HSF1 is associated with a dismal prognosis in human iCCA and is vital for rodent cholangiocarcinogenesis. Thus, targeting HSF1 might represent an innovative and attractive therapy against this deadly disease.

## Materials and methods

### Constructs and reagents

The plasmids used in the study, including pT3-EF1α-MYC-NICD1, pT3-EF1αYAPS127A, pT3-EF1αTAZS89A, pT3-EF1α-HAmyr–AKT, pT3-EF1α-V5-HSF1dn, pCMV empty vector, and pCMV/sleeping beauty transposase, have been previously described in detail [19, 21–24]. Plasmids were extracted using the Endotoxin Free Prep Kit (Sigma-Aldrich, St. Louis, MO, USA).

### Human tissue specimens

One hundred eighty-six human iCCA tissue samples from surgical resections were collected at the Institutes of Pathology of the Universities of Regensburg (Regensburg, Germany) and Greifswald (Greifswald, Germany). The clinicopathological data of the patients are reported in Table 1. The study was conducted according to the guidelines of the Declaration of Helsinki and approved by the Clinical Research Ethics Committee of the Medical Universities of Regensburg (approval code 17–1015–101) and Greifswald (approval code: BB 67/10).

**Table 1:** Clinicopathological features of iCCA patients

Variables	
No. of patients	72
Male	47
Female	25
Age (years)	
< 60	23
≥ 60	49
Etiology	
HBV	14
HCV	10
Hepatolithiasis	5
PSC	3
NA	40
Liver cirrhosis	
Yes	21
No	51
Tumor differentiation	
Well	35
Moderately	20
Poorly	17
Tumor size (cm)	
< 5	53
> 5	19
Tumor number	
Single	58
Multiple	14
Prognosis	
Better (≥ 3 years)	25
Poorer (< 3 years)	47
Lymph node metastasis	
Yes	24
No	48
Lung metastasis	
Yes	15
No	57

*Abbreviations*: *NA* Not available, *PSC* Primary sclerosing cholangitis

### Mouse experiments

Wild-type female FVB/N mice were purchased from the Jackson Laboratory (Sacramento, CA). At six to eight weeks of age, mice were subjected to hydrodynamic gene delivery, as described previously [25], to induce iCCA formation. To determine the tumor potential of HSF1, 20 μg of pT3-EF1α-MYC-NICD1, pT3-EF1α-YAPS127A, or pT3-EF1α TAZS89A, combined with 20 μg of pT3-EF1α-HA-myr-AKT, either alone or in combination with 40 μg pT3-EF1α-V5-HSF1dn, were mixed with pCMV/sleeping beauty transposase at a ratio of 25:1 and injected into mice via the lateral tail vein. All mice used in the experiments were monitored continually and euthanized when they showed signs of sickness induced by the tumor burden, such as enlarged liver or health deterioration. Due to the slower tumor development in HSF1dn mice (see later), these mice were sacrificed at later time points compared to the control mice. Mice were maintained and monitored following protocols approved by the Committee for Animal Research at the University of California, San Francisco (San Francisco, CA, USA).

### RNA extraction and quantitative real-time reverse transcriptase-polymerase chain reaction (qRT-PCR)

Total mRNA from liver tissues was extracted using the Quick RNA Miniprep kit (Zymo Research, Irvine, CA, USA). Subsequently, mRNA expression of the gene of interest was assessed by quantitative real-time polymerase chain reaction (qRT-PCR) using the validated Gene Expression Assay from Thermo Fisher Scientific (Waltham, MA, USA) for human *HSF1* (Hs00610436_m1). PCR reactions were performed with 100 ng of cDNA of the collected samples, using an ABI Prism 7000 Sequence Detection System with TaqMan Universal PCR Master Mix (Applied Biosystems). We applied the following cycling conditions: denaturation at 95 °C for 10 min, 40 cycles at 95 °C for 15 s, and then extension at 60 °C for 1 min. Quantitative values were calculated using the PE Biosystems Analysis software and expressed as N target (NT). NT = 2^−ΔCt^, where each sample’s ΔCt value was calculated by subtracting the average Ct value of *HSF1* from the average Ct value of the *β-Actin* gene (4333762T; Thermo Fisher Scientific).

### Cell lines, reagents, and treatments

The HuCCT1, KKU-M213, and KKU-M156 human iCCA cell lines, purchased from the Japanese Collection of Research Bioresources (JCRB) or the American Type Culture Collection (ATCC), were used for the experiments. Cell Lines Service (Eppelheim, Germany) performed cell line authentication. KKU-M156 and KKU-M213 cells were grown in Dulbecco’s modified Eagle medium (Gibco, Grand Island, NY, USA), whereas HuCCT1 cells were grown in RPMI 1640 medium (Gibco). Media were supplemented with 5% fetal bovine serum (Gibco), 100 mg/mL streptomycin, and 100 U/mL penicillin. Cells were cultured at 37 °C in a 5% CO_2_ humidified atmosphere. The mycoplasma-free status for the cell lines was recurrently checked with the PCR Mycoplasma Test Kit I (PromoCell, Heidelberg, Germany). KRIBB-11 (10 µM; MedChem Express) and ABT-263 (2,5 µM; MedChemExpress) were applied in the in vitro experiments. For gene silencing experiments, KKU-M156, HuCCT1, and KKU-M213 cell lines were transfected with siRNA against human *HSF1* (# 3297; siTOOLs Biotech GmbH, Planegg, Germany) or scrambled siRNA (# s4390846, negative control) (Thermo Fisher Scientific) using the Lipofectamine RNAiMAX Transfection Reagent (Thermo Fisher Scientific) according to the manufacturer’s protocol.

### Proliferation and apoptosis assays

The HuCCT1, KKU-M213, and KKU-M156 human iCCA cell lines were seeded in 96-well plates at a density of 10 × 10^4^ cells per well. Cell proliferation was evaluated in the cell lines at the 48-h time point using the BrdU Cell Proliferation Assay Kit (Cell Signaling Technology, Danvers, MA, USA). Following KRIBB-11 and/or ABT-263 treatment, cells were incubated with 1× bromodeoxyuridine (BrdU) for 2 h and fixed for 30 min at room temperature. The fixing solution was discarded, and cells were incubated with the BrdU mouse detection antibody for 1 h at room temperature. After washing, cells were stained with HRP-conjugated anti-mouse secondary antibody for 30 min at room temperature. After incubation with 3,3',5,5'-tetramethylbenzidine (TMB) substrate solution for 30 min at room temperature, stop solution was added, and the absorbance was measured at 450 nm. Apoptosis was determined at 48 h in iCCA cell lines using the Cell Death Detection Elisa Plus Kit (Roche Molecular Biochemicals, Indianapolis, IN, USA), following the manufacturer’s instructions. Cell line experiments were repeated at least three times in triplicate.

### Protein extraction and Western blot analysis

Human iCCA cell lines were homogenized in T-PER™ lysis buffer (Thermo Fisher Scientific) containing the 1x Halt™ Protease and Phosphatase Inhibitor Cocktail (Thermo Scientific) for 1 h on ice. Protein concentrations were determined using the colorimetric BioRad Protein Assay Dye Reagent Concentrate (500–0006, Bio-Rad, Hercules, CA, USA) with bovine serum albumin (BSA) as the standard. For Western blot analysis, protein lysates were denatured in 1× LDS Bolt™ sample buffer (B0007, Invitrogen) and 1× Bolt™ reducing agent (B00009, Invitrogen) by boiling for 5 min at 95 °C. Ten µL of proteins per lane were separated by SDS-PAGE on Bolt™ 4–12% Bis–Tris Mini Protein Gels (NW04125BOX, Invitrogen) in 1× Bolt™ MES SDS Running Buffer (B000202, Invitrogen) and transferred onto nitrocellulose membranes (iBlot™ 2 Mini/Regular Transfer Stacks, IB23001/2, Thermo Fisher Scientific) by electroblotting in the iBlot™ 2 Gel Transfer Device (IB21001, Thermo Fisher Scientific). Membranes were blocked in EveryBlot Blocking Buffer (12010020, Bio-Rad) and probed at + 4 °C overnight with the specific antibodies against HSF1 (#12972; 1:1000; Cell Signaling Technology), GAPDH (# sc-47724, 1:1000; Santa Cruz Biotechnology, Santa Cruz, CA), and β-actin (#ab20272; 1:5000; Abcam). Horseradish peroxidase-conjugated secondary antibody (1:10,000) was incubated for 1 h at room temperature, and blots were imaged with the Clarity Max ECL Western Blotting Substrate (Bio-Rad) using the ChemiDoc MP instrument (17001402, Bio-Rad).

### Immunohistochemistry

Human and mouse liver specimens were harvested, fixed in 10% formalin o/n at 4 °C, and embedded in paraffin. Hematoxylin and eosin (Thermo Fisher Scientific) staining was conducted using a standard protocol on liver sections. Specifically, antigen retrieval was performed in 10 mM sodium citrate buffer (pH 6.0) by heating in a microwave on high for 10 min, followed by a 20-min cool down at room temperature. After blocking with 5% goat serum and the Avidin–Biotin blocking kit (Vector Laboratories, Burlingame, CA, USA), the slides were incubated with the primary antibodies anti-HSF1 (1:100; HPA008888, Sigma-Aldrich, St. Louis, MO), V5-Tag (1:100; ab27671; Abcam), anti-cytokeratin 19 mouse-reactive (1:400; ab133496; Abcam), anti-cytokeratin 19 human-reactive (1:500; ab52625; Abcam), and anti-Ki-67 (1:800; 9449; Cell Signaling Technology, Danvers, MA) overnight at 4 °C. Slides were then subjected to 3% hydrogen peroxide for 10 min to quench endogenous peroxidase activity. Subsequently, the biotin-conjugated secondary antibody was applied at a 1:500 dilution for 30 min at room temperature. Reaction detection was achieved using the Vectastain ABC-Elite Peroxidase Kit (Vector Laboratories, # PK-6100) with ImmPACT DAB (Vector Laboratories, SK-4105) as the chromogen. Slides were counterstained with Mayer’s hematoxylin. The proliferation index was determined in human and mouse iCCA lesions to assess tumor proliferation by counting Ki-67 positive cells on at least 2000 tumor cells per sample.

### Seahorse mitochondrial respiration and glycolysis analyses

Seahorse assay on adherent cells was performed according to the manufacturer’s instructions (Agilent Technologies, Santa Clara, CA, USA). HuCCT1 and KKU-M156 (10 × 10^4^) cells per well were seeded into a Seahorse XFp Cell Culture 8 well Miniplate and cultured overnight at 37 °C and 5% CO_2_ for 24 h in standard culture medium. Two wells with medium only served as background correction. Experiments were conducted using three technical replicates per group. The next day, cells were treated with the IC_50_ of KRIBB-11 (10 µM final; MedChemExpress) or matched DMSO concentration in triplicates and incubated for 24 h at 37 °C and 5% CO_2_. One Seahorse XFp sensor cartridge per cell culture plate was hydrated with Seahorse XF Calibrant overnight at 37 °C in a CO2-free incubator. On the day of assay, the cells were washed and equilibrated with assay medium (Seahorse XF DMEM Medium pH 7.4, 10 mM glucose, 1 mM sodium pyruvate, and 2 mM L-glutamine) for 1 h at 37 °C in a CO_2_-free incubator and transferred to the Seahorse XF HS Mini Analyzer (Agilent Technologies). Injection ports were loaded either with i) 1.5 μM oligomycin (1.5 µM final; MCE), FCCP (HuCCT1, 2 µM final; MCE) and rotenone/antimycin A (Rot/AA, 0.5 µM final; Rot, MCE: AA, Biomol) for the Mitochondria Stress Test or with ii) Rot/AA (0.5 µM final) and 2-deoxy-D-glucose (2-DG; 50 mM final; Sigma-Aldrich) for the Glycolytic Rate Assay. Lastly, Hoechst 33342 (2 µg/µl final; Thermo Fisher Scientific) nuclei staining was injected per well and incubated for 10 min before fluorometric measurement (excitation, 355–20 nm; emission, 460 nm) using the FluoroStar Omega plate reader (BMG Labtech). Data were analyzed using the WAVE Pro 10.1.0.1 software (Agilent). The values were corrected to the background, normalized to the mean fluorescence intensity (MFI) per well, and a normalization scale factor of 10,000 was applied. To perform statistical analysis using GraphPad Prism 9.5.1. (GraphPad Prism Software Inc., USA-CA), data from three replicates of two independent experiments were used.

### Human cancer-associated fibroblast (hCAF) isolation

This study was performed following the approval of the local ethics committee, Istituto Tumori “Giovanni Paolo II” (Bari, Italy); protocol number: 145; date of release: March 2022. iCCA tissue specimens, derived from surgical resection, were immediately stored in MACS tissue storage solution (Miltenyi Biotec, Bergisch Gladbach, Germany) and processed. Upon receipt, biopsies of iCCA immediately after surgery were shredded into small pieces (1–2 mm) and stored in MACS tissue storage solution (Miltenyi Biotec, Bergisch Gladbach, Germany). Then, iCCA tissue specimens underwent enzymatic and mechanical digestion in HBSS solution with 50–200 U/mL collagenase Type IV (Thermo Fisher Scientific), 3 mM CaCl2, and Antibiotic–Antimycotic (Thermo Fisher Scientific) at 37 °C under rotation for 2 h (or longer, if necessary). The resulting cells were harvested, washed three times with HBSS by centrifugation, resuspended in IMDM with 20% FBS, and centrifuged at 500× g for 10 min. The resultant cell pellet, consisting of CAFs, was cultured in complete minimum essential medium (IMDM), a modified Dulbecco’s modified Eagle medium (DMEM) with 20% fetal bovine serum (FBS, Thermo Fisher Scientific) and Antibiotic–Antimycotic. iCCA hCAFs isolated from multiple patients were treated with vehicle, ABT-263, KRIBB-11, and the combination of the two drugs.

### Human intrahepatic cholangiocarcinoma patient-derived organoids culture and treatment

To collect iCCA patient-derived organoids (PDOs), after cell recovery, following iCCA tissue digestion, we used approximately 2 × 10^6^ cells for organoid culture, resuspended in 120 µL of liquefied growth factor reduced (GFR) Matrigel (Corning, Glendale, AZ, USA), creating a single droplet working on ice and seeded in a 24-well tissue culture plate, pre-warmed to 37 °C. Plates were incubated at 37 °C, 5% CO 2 in the cell culture incubator for 20 min to solidify Matrigel droplets; subsequently, we added the specific medium of the HepatiCult™ Organoid Kit (Human) (STEMCELL Technologies) for initiation and growth of organoids. When organoids became over-confluent, we broke up the droplets mechanically using a solution of PBS-EDTA 1 mM containing 1× TrypLe. Furthermore, the dissociation was implemented by incubating the plate for 20 min at 37 °C. Dissociated single cells were washed with HBSS (Thermo Fisher Scientific) and pelleted at 1200 rpm, 5 min, 4 °C. Briefly, 2 × 10 3 cells were resuspended in GFR matrigel and seeded in standard 96-well cell culture plates with complete organoid growth media. After 4–5 days post seeding, iCCA organoids, isolated from multiple patients, were treated with vehicle, ABT-263, KRIBB-11, and the combination of the two drugs. The drug-containing medium was changed every 2 days until the end of treatment. After 10 days of treatment, cell viability was assayed using the CellTiter 96® AQueous (MTS) (Promega, Tokyo, Japan).

### Statistical analysis

GraphPad Prism version 10.2.1 software (GraphPad Software Inc., La Jolla, CA, USA) and IBM SPSS version 26 software (IBM Corp., Armonk, NY, USA) evaluated statistical significance. The *p* values for TCGA data were obtained from the UALCAN analysis tool. The non-parametric Wilcoxon signed-rank test was used for paired sample comparison. Kaplan–Meier curves were evaluated using the log-rank test. Tukey’s multiple comparison test was applied for multiple comparisons. Two-tailed values of *P* < 0.05 were considered significant. Data are expressed as the mean ± SD or SEM.

## Results

### HSF1 expression is increased in human intrahepatic cholangiocarcinoma

To determine the role of HSF1 in human intrahepatic cholangiocarcinoma (iCCA), we assessed first its levels in this disease. Initially, *HSF1* mRNA levels in human cancers were investigated using data obtained from The Cancer Genome Atlas (TCGA) and the UALCAN Data Analysis Portal (http://ualcan.path.uab.edu/; accessed on May 30, 2024). *HSF1* mRNA expression was upregulated in various cancer types, including breast invasive carcinoma (BRCA), colon adenocarcinoma (COAD), head and neck squamous cell carcinoma (HNSC), and prostate adenocarcinoma (PRAD) among others (Supplementary Figure 1A). In cholangiocarcinoma (normal tissues: *n* = 9; tumor tissues: *n* = 36, of which 30 were iCCA), *HSF1* was significantly overexpressed (Supplementary Figure 1A; CHOL: cholangiocarcinoma; Supplementary Figure 1B; *p* = 1.46 × 10^–14^) with similar incidences in men (*p* = 2.32 × 10^–06^) and women (*p* = 4.98 × 10^–10^; Supplementary Figure 1C) and elevated expression for patients aged between 21 and 80 years (Supplementary Figure 1D; 21–40 years, *p* = 1.23 × 10^–07^; 41–60 years, *p* = 1.02 × 10^–07^; 61–80 years: *p* = 7.89 × 10^–08^) compared to that in normal tissue. In addition, the TCGA data showed augmented expression of *HSF1* in all tumor grades compared to normal tissue (Supplementary Figure 1E). However, the data were not significant, probably due to the small cohort of specimens analyzed (Supplementary Figure 1E). In contrast, increased *HSF1* levels characterized tumor stages 1, 2, and 4 (Supplementary Figure 1F; Normal vs. Stage 1, *p* = 3.03 × 10^–09^; Normal vs. Stage 2, *p* = 2.20 × 10^–05^; Normal vs. Stage 4, *p* = 7.68 × 10^–03^). Also, high *HSF1* expression was found in N0 and N1 nodal metastasis status (Supplementary Figure 1G; Normal vs. N0, *p* = 2.61 × 10^–12^; Normal vs. N1, *p* = 2.09 × 10^–02^), exhibiting a trend of increased *HSF1* expression toward N1 specimens. Furthermore, the survival time of patients with elevated expression of *HSF1* was not significantly shorter than that of patients with low or medium *HSF1* levels (Supplementary Figure 1H; *p* = 0.74).

**Fig. 1:**
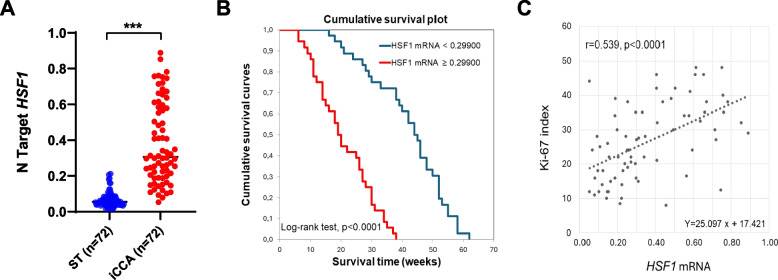
Upregulation of the *HSF1* gene in human intrahepatic cholangiocarcinoma specimens and correlation with survival and proliferation. **A** Quantitative real-time RT-PCR values of *HSF1* gene are significantly higher in the tumors (T) (*n* = 72) compared with corresponding non-tumorous counterparts (ST). **B** Kaplan-Meyer curve showing that *HSF1* mRNA levels inversely correlate with patients’ survival in this disease. **C** Linear regression analysis revealing that *HSF1* mRNA expression directly correlates with the tumors’ proliferation activity (as assessed with the Ki-67 index) in patients

Subsequently, to confirm and substantiate the findings from the TCGA dataset, we evaluated the mRNA levels of *HSF1* in 72 human iCCA samples and corresponding non-tumorous surrounding livers collected at the Universities of Regensburg and Greifswald, for which the survival data were available (Supplementary Material). In compliance with the TCGA dataset, quantitative real-time RT-PCR analysis revealed that *HSF1* mRNA levels were significantly higher in iCCA specimens than in paired non-tumorous tissues (*p* = 2.42654 × 10^–14^; Fig. 1A). Notably, when assessing the prognostic relevance of the gene, *HSF1* levels were inversely associated with patient survival time (*p* < 0.0001; Fig. 1B and Supplementary Material). Furthermore, we assessed the proliferation rate of the iCCA samples tested by evaluating the Ki-67 index. We found that the Ki-67 index positively and significantly correlates with *HSF1* levels (*r* = 0.539; *p* < 0.0001; Fig. 1C). In addition, the univariate analysis revealed a significant difference in survival for lymph node metastasis, lung metastasis, tumor number, and *HSF1* levels (Supplementary Material). Furthermore, multivariate analysis showed a significant difference in *HSF1* levels occurring only for survival, lymph node metastasis, and lung metastasis. Finally, correlation analysis unveiled that *HSF1* mRNA is negatively correlated with patient survival and positively correlated with lymph node and lung metastases (Supplementary Material).

Overall, the present data underline the overexpression of HSF1 in cholangiocarcinoma.

### HSF1 is upregulated in the various phases of human cholangiocarcinogenesis

**Fig. 2:**
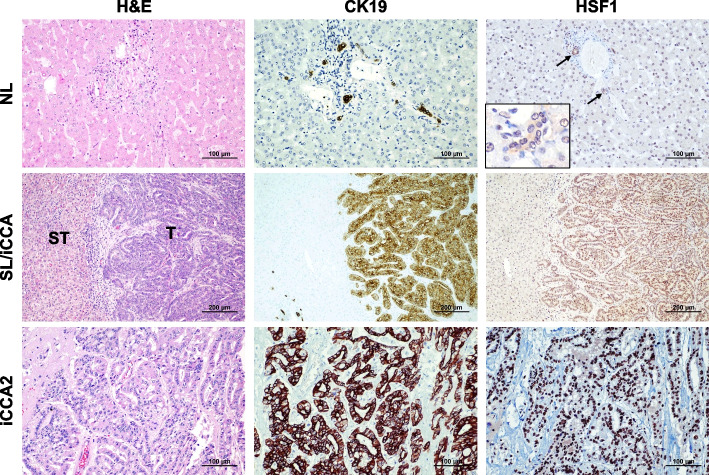
Representative immunohistochemistry patterns of HSF1 protein in human intrahepatic cholangiocarcinoma (iCCA; *n* = 186) and corresponding non-tumorous livers. The upper panels show a human normal liver (NL) displaying weak to moderate HSF1 immunoreactivity in hepatocytes and biliary cells (indicated by arrows). Staining of cholangiocytes (indicated by arrows) is better appreciable in the inset. Middle panels show the immunohistochemical staining of HSF1 in a non-tumorous surrounding tissue and one human iCCA specimen. The staining pattern for HSF1 is significantly more pronounced in the tumor compartment (T) compared with the neighboring non-tumorous surrounding tissue (ST), which exhibits faint HSF1 staining. Lower panels depict a second tumor (iCC2), characterized by intense nuclear HSF1 immunoreactivity. CK19 staining was used as a biliary marker. Abbreviation: H&E, hematoxylin and eosin staining. Original magnifications: 200x in the upper and lower panels, 100x in the middle panels, and 400x in the inset. Scale bar: 100 µm in the top and low panels, 200 µm in the middle panels

**Fig. 3:**
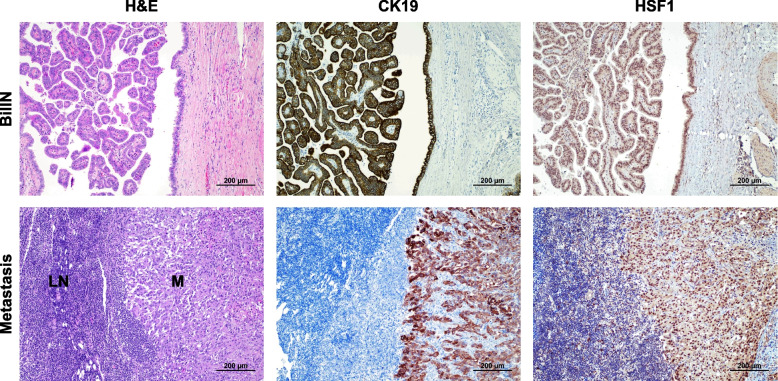
HSF1 is upregulated in human intrahepatic cholangiocarcinoma preinvasive lesions and metastases. Upper panels: Representative immunohistochemistry of HSF1 protein in a preinvasive lesion (one biliary intraepithelial neoplasia or BilIN specimen is shown here). The lesion exhibits pronounced nuclear immunoreactivity for HSF1. CK19 staining was used as a biliary marker. Lower panels: an iCCA lymph node metastasis displaying pronounced HSF1 nuclear immunolabeling in the tumor metastasis (M) and scattered nuclear positiveness in the lymph node (LN) compartment. CK19 staining was used as a biliary marker. Original magnification: 100x; scale bar: 200 μm. Abbreviations: H&E, hematoxylin and eosin staining

To further substantiate the overactivation of HSF1 in iCCA, the HSF1 protein levels were evaluated in a vast collection of human iCCA specimens (*n* = 186) by immunohistochemistry (Fig. 2). Robust nuclear immunoreactivity for HSF1 was ubiquitously observed in iCCA lesions. In contrast, weak HSF1 nuclear immunolabeling characterized hepatocytes from normal livers and surrounding non-neoplastic livers (ST). Furthermore, moderate cytoplasmic and nuclear staining for HSF1 was evident in normal cholangiocytes. In addition, pronounced nuclear immunoreactivity for HSF1 was detected in the totality of preinvasive lesions (*n* = 10, consisting of 6 intra-ductal papillary biliary neoplasms, IPBN, and 4 biliary intraepithelial neoplasias, BilIN) (Fig. 3). Moreover, pronounced nuclear accumulation of HSF1 characterized the iCCA metastases (*n* = 10, consisting of 4 lymph node and 6 peritoneal metastases (Fig. 3).

The present data indicate that HSF1 is ubiquitously induced in the various phases of human intrahepatic cholangiocarcinogenesis.

### Inactivation of HSF1 significantly delays cholangiocarcinogenesis in mice

**Fig. 4:**
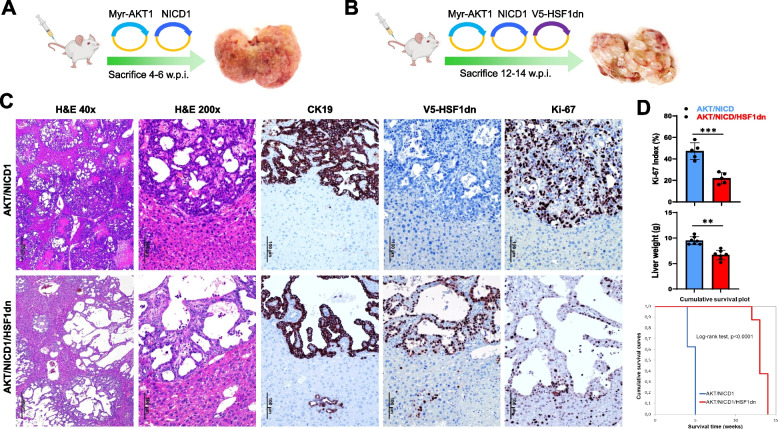
Suppression of HSF1 activity delays cholangiocarcinogenesis and reduces the proliferation and aggressiveness of AKT/NICD1 mouse lesions. **A**, **B** Study design. Briefly, wild-type FVB/N mice were subjected to hydrodynamic tail vein injection of either AKT/NICD1/pT3 (control) or AKT/NICD1/HSF1dn plasmids. HSF1dn is the dominant-negative form of the HSF1 transcription factor, whose overexpression effectively inhibits the endogenous HSF1 activity. **C** While liver lesions from AKT/NICD1 mice consisted of invasive and proliferative (as assessed by positive immunoreactivity for Ki-67) intrahepatic cholangiocarcinomas (upper panels), cystic lesions (denominated cystic adenomas and cystic adenocarcinomas) with low proliferation rate occupied the liver parenchyma of AKT/NICD1/HSF1dn mice. As expected, V5-tagged-HSF1dn staining was observed only in AKT/NICD1/HSF1dn mice. **D** Liver weight and proliferative activity were significantly higher in AKT/NICD1 mice than in AKT/NICD1/HSF1dn mice. Moreover, the survival curve showed significantly longer survival of AKT/NICD1/HSF1dn mice. Student’s *t-*test: ***, *P* < 0.0001; **, *P* < 0.001. Original magnifications: 40x and 200x; scale bar: 500 μm in 40x and 100 μm in 200x. Abbreviations: H&E, hematoxylin and eosin staining

Next, we determined the requirement of HSF1 for intrahepatic biliary carcinogenesis in various mouse models of iCCA that we have previously developed. First, to assess the relevance of HSF1 in AKT/NOTCH1-dependent cholangiocarcinogenesis, we overexpressed a myristoylated/activated form of AKT1 (myr-AKT1) and an activated/cleaved form of Notch1 (NICD1) while simultaneously inactivating HSF1 in the hepatocytes. To achieve this goal, we hydrodynamically transfected FVB/N mice with myr-AKT1, NICD1, and a dominant negative form of HSF1 [21] (HSF1dn; these mice will be referred to as AKT/NICD1/HSF1dn mice; *n* = 5). The dominant negative form of HSF1 inhibits the activity of the endogenous HSF1 [21]. As a control, we hydrodynamically transfected FVB/N mice with AKT1 and NICD1 (AKT/NICD1 mice; *n* = 5) (Fig. 4A, B). All AKT/NICD1 mice showed an enlarged liver with a consequent increase in liver weight compared to control mice. AKT/NICD1 mice rapidly deteriorated and required euthanasia by 4 to 5 weeks post hydrodynamic injection due to high tumor burden (Fig. 4C, D) in accordance with previous findings [22]. Macroscopically, AKT/NICD1 livers were cover by small nodular formations (Fig. 4A). Histologically, most of the liver parenchyma of AKT/NICD1 mice was occupied by numerous, often colliding, highly invasive iCCA lesions, often forming small ducts. Most tumor lesions exhibited additional signs of malignancy, including necrosis, high mitotic activity, and diffuse infiltration of the surrounding liver parenchyma (Fig. 4C, Supplementary Figure 2A), in accordance with previous reports [22]. In contrast, suppression of HSF1 by HSF1dn injection significantly slowed down tumorigenesis, and AKT/NICD1/HSF1dn mice were sacrificed significantly later, by 13–14 weeks post-injection (Fig. 4B, D). Of note, cholangiocellular tumors developed in AKT/NICD1/HSF1dn mice appeared less aggressive and resembled more closely the tumor lesions occurring in mice injected with NICD1 alone [22]. Indeed, lesions from AKT/NICD1/HSF1dn mice mainly consisted of low-proliferating and large cystic lesions filled with fluid, classified as cystadenomas and cystadenocarcinomas (Fig. 4C, Supplementary Figure 2B). However, a few invasive iCCA could also be observed in AKT/NICD1/HSF1dn livers. Macroscopically, the large fluid-filled cysts induced a pronounced “bubble-like” phenotype to AKT/NICD1/HSF1dn livers (Fig. 4B). Moreover, as assessed by the Ki-67 index, proliferation was significantly lower in AKT/NICD1/HSF1dn tumor lesions (Fig. 4D).

**Fig. 5:**
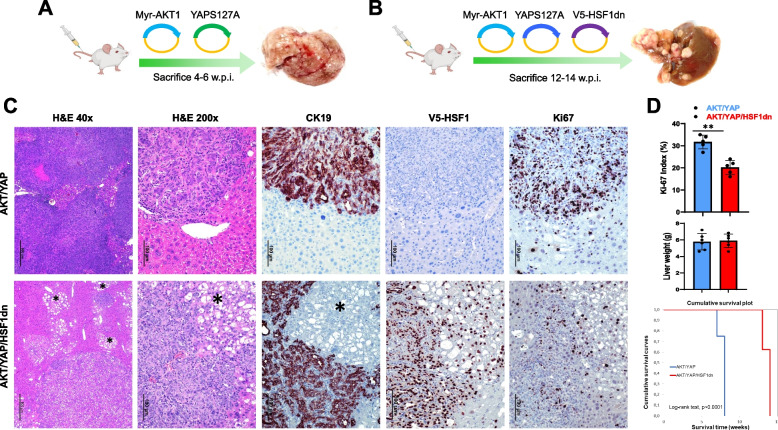
Suppression of HSF1 activity delays cholangiocarcinogenesis and reduces the proliferation and aggressiveness of AKT/YAP mouse lesions. **A**, **B** Study design. Briefly, wild-type FVB/N mice were subjected to hydrodynamic tail vein injection of either AKT/YAPS127A/pT3 (control; AKT/YAP mice) or AKT/YAPS127A/HSF1dn (AKT/YAP/HSF1dn mice) plasmids. In particular, HSF1dn is the dominant-negative form of the HSF1 transcription factor, whose overexpression effectively inhibits the endogenous HSF1 activity. **C** Liver lesions from AKT/YAP mice consisted of invasive and proliferative (as assessed by positive immunoreactivity for Ki-67) intrahepatic cholangiocarcinomas (upper panels). In contrast, neoplastic lesions from AKT/YAP/HSF1dn consisted of both hepatocellular, clear-cell (indicated by an asterisk), and cholangiocellular lesions with low proliferation rates. The hepatocellular lesions were CK19-negative, whereas the cholangiocellular lesions were CK19-positive. As expected, V5-tagged-HSF1dn staining was observed only in AKT/YAP/HSF1dn mice. **D** While liver weight was equivalent in the two mouse cohorts, the proliferative activity was significantly higher in AKT/YAP mice than in AKT/YAP/HSF1dn mice. Moreover, the survival curve showed significantly longer survival of AKT/YAP/HSF1dn mice. Student’s *t*-test: **, *P* < 0.001. Original magnifications: 40x and 200x; scale bar: 500 μm in 40x and 100 μm in 200x. Abbreviations: H&E, hematoxylin and eosin staining

**Fig. 6:**
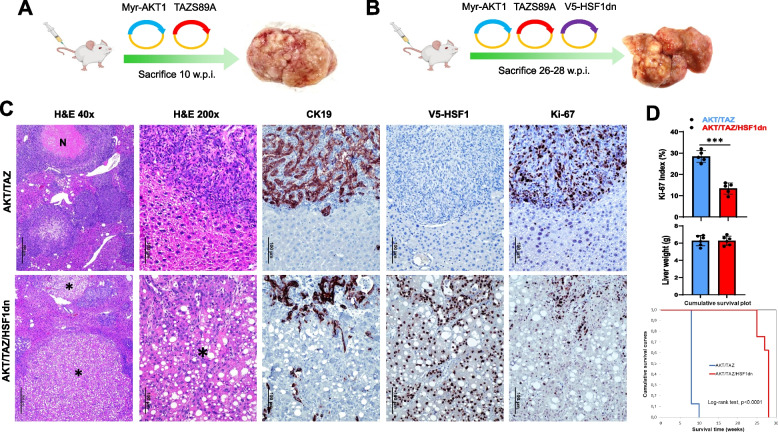
Suppression of HSF1 activity delays cholangiocarcinogenesis and reduces the proliferation and aggressiveness of AKT/TAZ mouse lesions. **A**, **B** Study design. Briefly, wild-type FVB/N mice were subjected to hydrodynamic tail vein injection of either AKT/TAZS89A/pT3 (control; AKT/TAZ mice) or AKT/TAZS89A/HSF1dn (AKT/TAZ/HSF1dn mice) plasmids. HSF1dn is the dominant-negative form of the HSF1 transcription factor, whose overexpression effectively inhibits the endogenous HSF1 activity. **C** Liver lesions from AKT/TAZ mice consisted of invasive and proliferative (as assessed by positive immunoreactivity for Ki-67) intrahepatic cholangiocarcinomas (upper panels) with frequent necrotic areas (N). In contrast, neoplastic lesions from AKT/TAZ/HSF1dn consisted of both hepatocellular, clear-cell (indicated by asterisks), and cholangiocellular lesions with low proliferation rates. The hepatocellular lesions were CK19-negative, whereas the cholangiocellular lesions were CK19-positive. As expected, V5-tagged-HSF1dn staining was observed only in AKT/TAZ/HSF1dn mice. **D** While liver weight was equivalent in the two mouse cohorts, the proliferative activity was significantly higher in AKT/TAZ mice than in AKT/TAZ/HSF1dn mice. Moreover, the survival curve showed significantly longer survival of AKT/TAZ/HSF1dn mice. Student’s *t*-test: **, *P* < 0.0001. Original magnifications: 40x and 200x; scale bar: 500 μm in 40x and 100 μm in 200x. Abbreviations: H&E, hematoxylin and eosin staining

Subsequently, we applied the same approach to two additional mouse models of iCCA that we previously established: the AKT/YAP and the AKT/TAZ [23, 24]. Specifically, mice were injected with myr-AKT1, activated forms of YAP (YAP^S127A^; AKT1/YAP mice; *n* = 5) or TAZ (TAZ^S89A^; AKT1/TAZ mice; *n* = 5), and HSF1dn. As a control, we hydrodynamically transfected FVB/N mice with either AKT/YAP (*n* = 5), or AKT/TAZ (*n* = 5) (Figs. 5, 6, Supplementary Figures 3 and 4). Similar to that described in the AKT/NICD1 model, cholangiocarcinogenesis was significantly delayed in AKT/YAP and AKT/TAZ mice depleted of HSF1 (AKT/YAP/HSF1dn and AKT/TAZ/HSF1dn) compared with AKT/YAP and AKT/TAZ mice retaining an intact HSF1 (Figs. 5, 6, Supplementary Figures 3 and 4). Macroscopically, the livers of AKT/YAP- and AKT/TAZ-injected mice exhibited mainly a solid appearance. In contrast, numerous whitish or yellowish nodular lesions resembling the aspect of hepatocellular carcinomas characterized AKT/YAP/HSF1dn- and AKT/TAZ/HSF1dn-injected livers (Figs. 5A and 6A). At the histological level, AKT/YAP and AKT/TAZ tumors consisted almost exclusively of pure cholangiocarcinoma lesions, although scattered lipid-rich hepatocellular preneoplastic lesions could be detected in the two rodent cohorts, with a predominance in AKT/YAP livers (Figs. 5C and 6C, upper panels, and Supplementary Figures 3A and 4A). In striking contrast, large areas of AKT/YAP/HSF1dn and AKT/TAZ/HSF1dn liver parenchyma were occupied by hepatocellular preneoplastic and neoplastic lesions with a clear cell phenotype due to lipid accumulation (Figs. 5C and 6C, and Supplementary Figures 3B and 4B). These lesions have been extensively described before and are typical of AKT-overexpressing livers [26]. CK19-positive cholangiocellular lesions were still present in AKT/YAP/HSF1dn and AKT/TAZ/HSF1dn livers, but occupied only a minor part of the parenchyma (Figs. 5C and 6C, and Supplementary Figures 3B and 4B).

Overall, the present data indicate that HSF1 inactivation significantly delays cholangiocarcinogenesis in several mouse models of iCCA by reducing proliferation and inducing hepatocellular differentiation.

### Genetic and pharmacologic HSF1 inactivation restrains intrahepatic cholangiocarcinoma growth and rewires cell metabolism in vitro

**Fig. 7:**
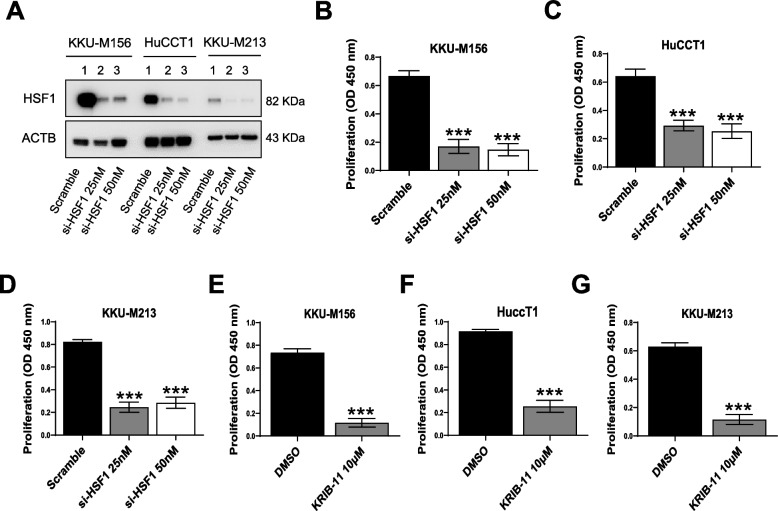
Effect of HSF1 inhibition on the growth of intrahepatic cholangiocarcinoma cell lines. **A** Inhibition of HSF1 via specific small-interfering RNA in KKU-M156, HuCCT1, and KKU-M213 human intrahepatic cholangiocarcinoma (iCCA) cell lines, as assessed by Western blot analysis. **B**-**D** BrdU incorporation assay was conducted on **B** KKU-M156, **C** HuCCT1, and **D** KKUM-213 cells. Analysis was conducted 48 h after siRNA administration. Two siRNA concentrations (25 and 50 nm) were applied. Scramble-treated cells served as control. **E**–**G** KKU-M156, HuCCT1, and KKUM-213 cells were also treated for 48 h with 10 µM KRIBB-11, an HSF1 inhibitor, and BrdU incorporation was assessed. DMSO-treated cells served as controls. The values in optical densities (OD) at 450 nm are presented. All results are expressed as mean ± SD of three independent experiments in triplicate. For statistical analysis, Tukey’s multiple comparisons test was performed (*** *p* < 0.0001, vs. scramble and DMSO). Abbreviation: si-HSF1, small-interfering RNA against HSF1

Next, we determined the effect of suppressing HSF1 in iCCA cells. For this purpose, *HSF1* was silenced in KKU-M213, HuCCT1, and KKU-M156 iCCA cell lines using a pool of small interfering RNA (siRNA). Effective downregulation of HSF1 in the three cell lines was demonstrated by Western blot analysis (Fig. 7A) and quantitative real-time RT-PCR (not shown). At the cellular level, the knockdown of *HSF1* resulted in a significant reduction of proliferation (Fig. 7B-D) and a limited rise in apoptosis (Supplementary Figure 5A-C) of the three cell lines compared with cells treated with scrambled siRNA. Similar cell growth and apoptosis modifications were detected using the HSF1 inhibitor KRIBB-11, which was used at his IC50 (10 µM). Indeed, the three cell lines treated with KRIBB-11 displayed low proliferation and higher apoptosis than the same cells subjected to DMSO administration, with a significantly less pronounced effect on apoptosis. Next, we determined whether an apoptosis inducer could augment the limited pro-apoptotic effect of KRIBB-11. For this purpose, the three iCCA cell lines were treated with KRIBB-11, either alone or in combination with the potent pro-apoptosis drug ABT-263 (navitoclax), a Bcl-xL/Bcl2/Bcl-w inhibitor. While both KRIBB-11 (10 µM) and ABT-263 (up to 50 µM, not shown) alone had either minor or no effects on apoptosis, their combination promoted a strong induction of cell death in the three cell lines (Supplementary Figure 6A-C). Notably, no synergistic effect of the two drugs on proliferation was detected (Supplementary Figure 6D-F).

**Fig. 8:**
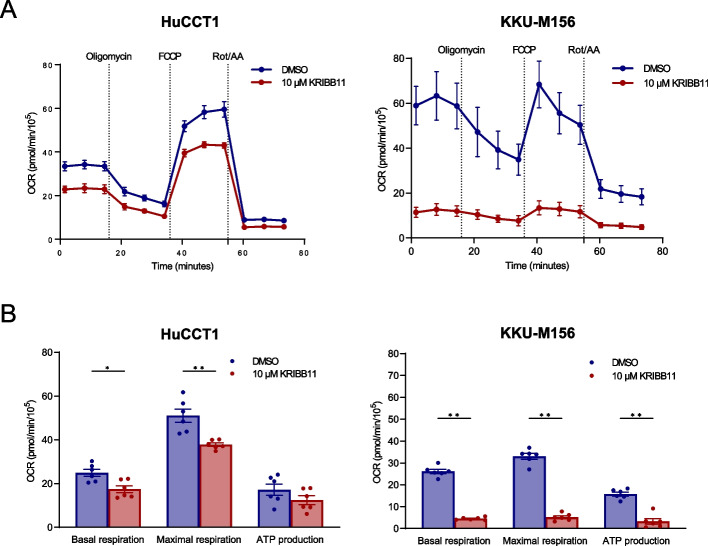
The HSF1 inhibitor KRIBB11 decreases the mitochondrial bioenergetics in cholangiocarcinoma cell lines. **A** Cell Mito Stress test profiles of human HuCCT1 and KKU-M156 cell lines treated with 10 µM KRIBB11 and matching DMSO concentration for 24 h. Seahorse XF Cell Mito Stress Tests (Agilent) were performed employing serial injections of oligomycin, FCCP, and Rot/AA; Hoechst 33342 was injected last for nuclei staining required for normalization. OCR was measured in real-time using the Seahorse XF HS mini analyzer. **B** Changes in basal respiration, maximal respiration and ATP production. Data were background corrected and normalized to the mean fluorescent intensity per well; a factor 10^5^ was applied. Graphs depict the mean ± SEM of two independent experiments each performed in technical triplicates (multiple Mann–Whitney tests; *p < 0.05; **p < 0.01). Abbreviations: OCR, oxidative consumption rate (in pmol/min); FCCP, carbonyl cyanide-p-trifluoromethoxyphenylhydrazone; Rot/AA, rotenone/antimycin A

**Fig. 9:**
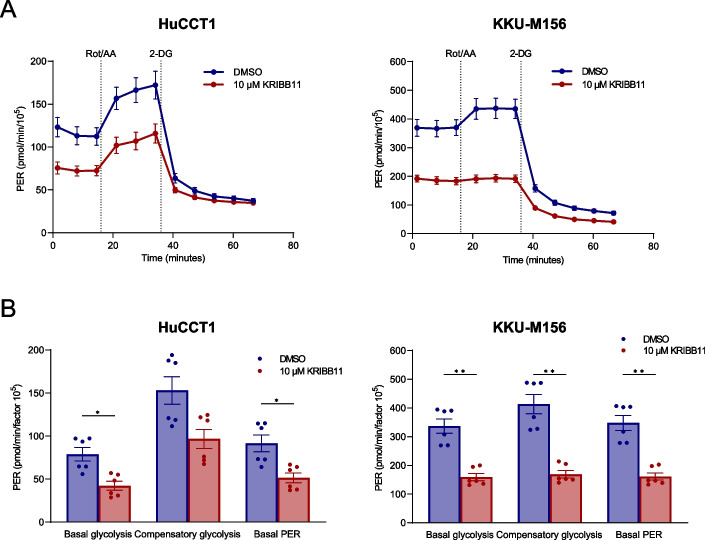
The HSF1 inhibitor KRIBB11 reduces the glycolytic metabolism of cholangiocarcinoma cell lines. **A** Glycolytic rate profiles of human HuCCT1 and KKU-M156 cell lines treated with 10 µM KRIBB11 and matching DMSO concentration for 24 h. Seahorse XF Glycolytic Rate assays (Agilent) were performed employing serial injections of Rot/AA, and 2-DG and Hoechst 33342 for nuclei staining; the PER reflecting the glycolytic function was measured using the Seahorse XF HS mini analyzer. **B** Changes in basal glycolysis, compensatory glycolysis, and basal PER. Data were background corrected and normalized to cell number as determined by Hoechst 33342 nuclei staining; a factor of 10^5^ was applied. Graphs depict the mean ± SEM of two independent experiments each performed in technical triplicates (multiple Mann–Whitney tests; *p < 0.05). Abbreviations: PER, proton efflux rate (in pmol/min); Rot/AA, rotenone/antimycin A; 2-DG, 2-deoxy-D-glucose

Subsequently, based on the previous evidence indicating a critical role of HSF1 in tumor metabolism, we investigated mitochondrial respiration and glycolysis in the HuCCT1 and KKU-M156 iCCA cell lines following KRIBB-11 administration using the Seahorse Assay. Concerning the mitochondrial bioenergetics, KRIBB-11 (10 µM) decreased basal and maximal mitochondrial respiration significantly compared with solvent alone in the two cell lines; a similar trend was detected for ATP production, although it did not reach statistical significance in HuCCT1 cells (Fig. 8A, B). In addition, KRIBB-11 administration significantly reduced basal glycolysis, compensatory glycolysis, and protein efflux rate in both iCCA cell lines (Fig. 9A, B).

Taken together, the present data indicate that suppression of HSF1 decreases cell growth and affects the metabolism of iCCA cells in vitro.

### HSF1 inactivation reduces the growth properties of intrahepatic cholangiocarcinoma cancer-associated fibroblasts in vitro

**Fig. 10:**
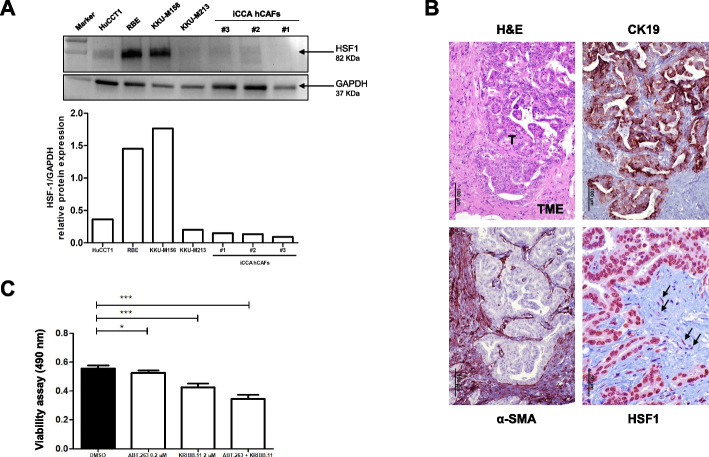
The HSF1 inhibitor KRIBB-11 reduces the growth in vitro of human cancer-associated fibroblasts. **A** Levels of HSF1 in human HuCCT1, RBE, KKU-M156, and KKU-M213 intrahepatic cholangiocarcinoma (iCCA) cell lines, and in human cancer-associated fibroblasts (hCAFs), as determined by Western blot analysis. GAPDH was used as a housekeeping protein and protein values of each sample are reported below. **B** Representative immunohistochemistry showing immunoreactivity for HSF1 in the tumor (T) and the tumor microenvironment (TME) compartments. Arrows indicate fibroblasts showing moderate immunolabeling for HSF1 protein. Alpha-smooth-muscle (α-SMA) staining was used as a tumor stroma marker, while CK19 staining was used as a biliary marker. Original magnification: 200x for H&E, CK19, and α-SMA; 400x for HSF1; scale bar: 100 μm for H&E, CK19, and α-SMA; 50 μm for HSF1. Abbreviations: H&E, hematoxylin and eosin staining. **C** The co-treatment with ABT-263 and KRIBB-11 inhibits the cell viability in iCCA hCAFs. Effect of ABT-263, KRIBB-11, and their co-administration on the viability of iCCA hCAFs, grown for 72 h in culture medium supplemented with 10% FBS. Note the higher anti-growth effects of the two combined drugs than single treatments. Experiments were repeated three times in triplicate. *P < 0.05 and ***P < 0.001, as calculated with a One-way ANOVA test

Mounting evidence indicates that HSF1 positively influences the tumor microenvironment by favoring the growth of cancer-associated fibroblasts (CAFs) [27–30]. Thus, we determined whether the same applies to iCCA. For this purpose, human primary iCCA CAFs (hCAFs), isolated from tumor biopsies, were tested for HSF1 levels. Western blot analysis revealed an heterogeneous expression of HSF1 in human iCCA cell lines and hCAFs. Specifically, the highest levels of HSF1 characterized KKU-M156 and RBE iCCA cells, whereas lower levels (but still higher than in hCAFs) were found in HuCCT1 tumor cells. Similar lower levels of HSF1 were detected in KKU-M213 iCCA cells and the three hCAFs (Fig. 10A). On the other hand, HSF1 levels were more elevated in tumor cells than hCAFs in human iCCA specimens, as detected by immunohistochemistry (Fig. 10B). Subsequently, we treated the hCAFs with ABT-263 and KRIBB-11. Notably, ABT-263 and KRIBB-11 anti-growth effects were achieved at lower concentrations than in monolayer iCCA cell lines. Specifically, ABT-263 treatment (at 0,2 µM) significantly inhibited cell viability after 72 h, in accordance with a previous report [31]. Also, the cell challenge with the small molecule KRIBB-11 induced a more pronounced reduction of cell viability in these cell cultures. Furthermore, combination treatment with both drugs synergistically suppressed cell viability of iCCA hCAFs compared to untreated cells (Fig. 10C).

Thus, KRIBB-11 reduces the cell viability of hCAFs, which is further decreased by the combination with ABT-263.

### KRIBB-11 and ABT-263 inhibit the cell viability of iCCA organoids

**Fig. 11:**
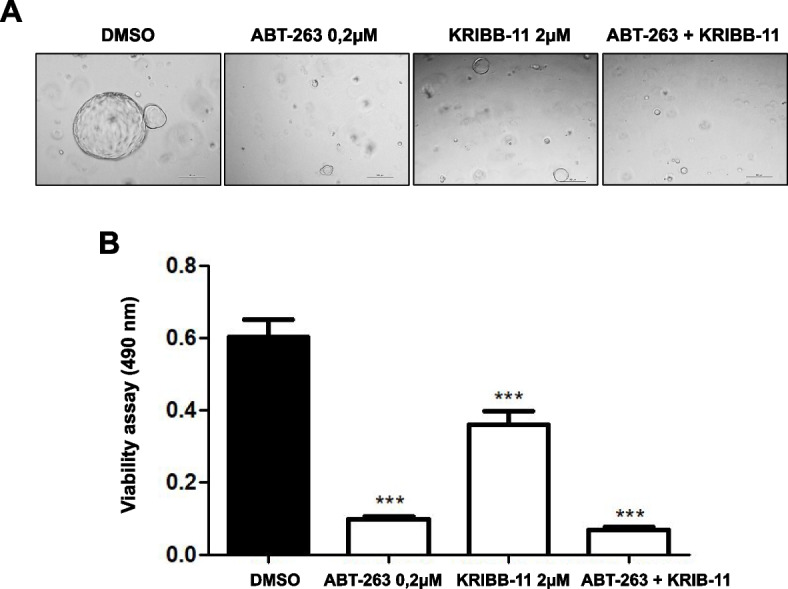
The HSF1 inhibitor KRIBB-11 and the apoptosis inducer ABT-263 reduce the viability of iCCA organoids. **A**, **B** Effect of ABT-263, KRIBB-11, and their combination on the viability of iCCA organoids, treated for 10 days in the specific medium of the hepatic organoid. Experiments were repeated three times in triplicate. ***P < 0.001, as calculated with a One-way ANOVA test

Finally, different iCCA organoid preparations were treated with vehicle, ABT-263, KRIBB-11, and the combination of the two drugs (Fig. 11). Similar to that described for hCAFs, both drugs were effective on organoid growth at lower levels than in monolayer cell lines. In particular, ABT-263 treatment (0,2 µM) drastically inhibited organoid cell viability. KRIBB-11 treatment also significantly reduced cell viability, but it was less remarkable compared to the effect of the first drug. When we simultaneously subjected the organoids to therapy with both drugs, a reduction in cell viability induced by KRIBB-11 was slightly but significantly improved by the presence of ABT-263 in the medium (Fig. 11A, B).

## Discussion

Intrahepatic cholangiocarcinoma (iCCA) is an aggressive disease of the biliary tree characterized by asymptomatic onset, limited therapeutic options, and poor prognosis. Despite its higher prevalence in Southeast Asia, iCCA incidence constantly increases in Western countries and is becoming a significant health concern worldwide. Therefore, innovative and more effective therapeutic options are urgently needed to make this disease curable [1–8].

In the present investigation, we have evaluated the pathogenetic role of HSF1, the director of cell response to stressors [9–11], in iCCA. We revealed that levels of HSF1 are upregulated in the various phases of cholangiocarcinogenesis, from preinvasive to metastatic lesions, implying the requirement of HSF1 for both iCCA development and progression. Furthermore, in agreement with a previous report [17], we found that levels of HSF1 inversely correlate with the length of patients’ survival in this aggressive disease. Moreover, in vitro and in vivo approaches indicated that inactivation of HSF1 is highly detrimental to the growth of iCCA cell lines, cancer-associated fibroblasts, tumor organoids, and biliary tumors developed in mice. Therefore, the present data imply the pathogenetic relevance of HSF1 in iCCA, affecting the cancer cell compartment and the tumor microenvironment.

The observation of ubiquitous HSF1 activation and the finding that inhibition of HSF1 significantly reduces tumor cell proliferation unravels HSF1 as a potentially relevant target for treating this disease. We also found a synergistic effect when combining the HSF1 inhibitor KRIBB1 with the Bcl-xL/Bcl2/Bcl-w suppressor and apoptosis inducer ABT-263 in monolayer iCCA cell lines, resulting in massive apoptosis. The synergistic effect of the two drugs was less remarkable in CAFs and patient-derived organoids, presumably due to the high susceptibility of the organoid and hCAFs used for the analysis to the anti-growth action of KRIBB1 and ABT-263 drugs administered alone. Further studies using in vivo models are necessary to establish more precisely the potency of KRIBB-11 alone and in association with ABT-263.

Besides biliary tumor cells, the present investigation shows that HSF1 affects the growth of iCCA CAFs, since HSF1 inhibition significantly reduced the vitality of this stromal component. The data agree with those described in other tumor types [27–30]. For instance, it has been shown that HSF1 is frequently activated in CAFs from various tumor entities, including breast and esophageal cancers [32, 33]. There are presumably multiple mechanisms whereby HSF1 positively influences and reshapes the tumor microenvironment. On the one hand, HSF1 induces a transcriptional program in CAFs that differs from that of adjacent cancer cells [27]. On the other hand, HSF1 protects tumor cells from the excess of substances and bioactive molecules produced by the tumor microenvironment [29, 30]. Furthermore, HSF1 seems to be involved in drug resistance of tumor cells mediated by macrophages [34–36]. In particular, overexpression of HSF1 by tumor-associated macrophages (TAM) is associated with resistance to chemotherapy in lung cancer cells and tumor xenografts [34]. Furthermore, it has been demonstrated that HSF1 produced by hepatocellular carcinoma cells promotes the functional crosstalk of tumor cells with TAM [35]. Thus, experiments should be conducted in complex systems including tumorous cells and non-tumorous supporting cells (CAFs, immune cells) to generate a more comprehensive picture of the effects of HSF1 suppression on cholangiocarcinogenesis in vivo. Using HSF1 knockout mice will help better elucidate the role of HSF1 in the iCCA microenvironment.

Regarding the HSF1 tumorigenic potential in vivo, we discovered that suppression of HSF1 delayed biliary tumor development in AKT/NICD1, AKT/YAP, and AKT/TAZ mice. Notably, tumors in the three models injected with HSF1dn appeared less aggressive at the histopathological level, exhibited a lower proliferation rate than the corresponding lesions in the mice with an active HSF1, and displayed some differentiation features toward the hepatocellular lineage. Thus, HSF1 affects multiple traits of the tumor cell, not limited to tumor proliferation. The mechanisms whereby HSF1 drives tumor development toward a more hepatocellular-like phenotype remain obscure and could be multiple. For instance, HSF1 could transcriptionally suppress genes involved in the hepatocellular lineage, while its inactivation might relieve their expression. On the other hand, we cannot exclude that malignant cholangiocytes rely on HSF1 more than neoplastic hepatocyte-like cells, with the consequent predominance of hepatocellular lesions in AKT/NICD, AKT/YAP, and AKT/TAZ livers deprive of an active HSF1.

The pleiotropic role of HSF1 in the aforementioned iCCA models agrees with the finding that HSF1 regulates a plethora of downstream effectors, as shown in yeast, where HSF1 transcriptionally controls more than 3% of the entire genome [37]. In accordance with this previous observation, we revealed that HSF1 inactivation by KRIBB-11 leads to decreased mitochondrial respiration and glycolytic activities of iCCA cells, further underscoring the multifaceted function of HSF1 on cell behavior. High-throughput analysis, such as transcriptomics and proteomics, should be applied to identify the HSF1 targets responsible for the various effects observed in the iCCA models analyzed.

Another intriguing discovery of the study was that HSF1 depletion delayed, but did not suppress, cholangiocarcinogenesis in the three mouse models. These data differ from the complete inhibition of liver tumorigenesis detected in mice overexpressing AKT or c-Myc alone [19, 20], implying compensatory mechanisms allowing tumor cell growth in conditions of HSF1 depletion. Identifying the critical players responsible for the survival of cancer cells depleted of HSF1 will be highly helpful for innovative therapies combining HSF1 inhibitors with other drugs.

In summary, we revealed that HSF1 is ubiquitously induced in human iCCA lesions, and HSF1 suppression is detrimental to the growth of malignant cholangiocytes and cancer-associated fibroblasts. Thus, pharmacological targeting of HSF1 in combination with other inhibitors might represent an innovative therapeutic strategy against this deadly disease.

## Supplementary Information

Supplementary Material 1.

Supplementary Material 2.

Supplementary Material 3.

Supplementary Material 4.

Supplementary Material 5.

Supplementary Material 6.

Supplementary Material 7.

## Data Availability

All data generated or analyzed during this study are included in this published article [and its supplementary information files].

## References

[1] Banales JM, Cardinale V, Carpino G, Marzioni M, Andersen JB, Invernizzi P, et al. Expert consensus document: cholangiocarcinoma: current knowledge and future perspectives consensus statement from the European Network for the Study of Cholangiocarcinoma (ENS-CCA). Nat Rev Gastroenterol Hepatol. 2016;13:261–80. 27095655 10.1038/nrgastro.2016.51

[2] Banales JM, Marin JJG, Lamarca A, Rodrigues PM, Khan SA, Roberts LR, et al. Cholangiocarcinoma 2020: the next horizon in mechanisms and management. Nat Rev Gastroenterol Hepatol. 2020;17:557–88. 32606456 10.1038/s41575-020-0310-z PMC7447603

[3] Ilyas SI, Khan SA, Hallemeier CL, Kelley RK, Gores GJ. Cholangiocarcinoma - evolving concepts and therapeutic strategies. Nat Rev Clin Oncol. 2018;15:95–111. 28994423 10.1038/nrclinonc.2017.157 PMC5819599

[4] El-Diwany R, Pawlik TM, Ejaz A. Intrahepatic cholangiocarcinoma. Surg Oncol Clin N Am. 2019;28:587–99. 31472907 10.1016/j.soc.2019.06.002

[5] Acher AW, Paro A, Elfadaly A, Tsilimigras D, Pawlik TM. Intrahepatic cholangiocarcinoma: a summative review of biomarkers and targeted therapies. Cancers. 2021;13:5169. 34680318 10.3390/cancers13205169 PMC8533913

[6] Greten TF, Schwabe R, Bardeesy N, Ma L, Goyal L, Kelley RK, et al. Immunology and immunotherapy of cholangiocarcinoma. Nat Rev Gastroenterol Hepatol. 2023;20:349–65. 36697706 10.1038/s41575-022-00741-4 PMC12468729

[7] Burris HA 3rd, Okusaka T, Vogel A, Lee MA, Takahashi H, Breder V, et al. Durvalumab plus gemcitabine and cisplatin in advanced biliary tract cancer (TOPAZ-1): patient-reported outcomes from a randomised, double-blind, placebo-controlled, phase 3 trial. Lancet Oncol. 2024;25:626–35. 38697156 10.1016/S1470-2045(24)00082-2

[8] Kelley RK, Ueno M, Yoo C, Finn RS, Furuse J, Ren Z, et al. Pembrolizumab in combination with gemcitabine and cisplatin compared with gemcitabine and cisplatin alone for patients with advanced biliary tract cancer (KEYNOTE-966): a randomised, double-blind, placebo-controlled, phase 3 trial. Lancet. 2023;401:1853–65. 37075781 10.1016/S0140-6736(23)00727-4

[9] Carpenter RL, Gökmen-Polar Y. HSF1 as a cancer biomarker and therapeutic target. Curr Cancer Drug Targets. 2019;19:515–24. 30338738 10.2174/1568009618666181018162117 PMC6472998

[10] Dai C. The heat-shock, or HSF1-mediated proteotoxic stress, response in cancer: from proteomic stability to oncogenesis. Philos Trans R Soc Lond B Biol Sci. 2018;373:20160525. 29203710 10.1098/rstb.2016.0525 PMC5717525

[11] Jiang S, Tu K, Fu Q, Schmitt DC, Zhou L, Lu N, et al. Multifaceted roles of HSF1 in cancer. Tumour Biol. 2015;36:4923–31. 26108999 10.1007/s13277-015-3674-x

[12] Xu M, Lin L, Ram BM, Shriwas O, Chuang KH, Dai S, et al. Heat shock factor 1 (HSF1) specifically potentiates c-MYC-mediated transcription independently of the canonical heat shock response. Cell Rep. 2023;42: 112557. 37224019 10.1016/j.celrep.2023.112557 PMC10592515

[13] Dai B, Gong A, Jing Z, Aldape KD, Kang SH, Sawaya R, et al. Forkhead box M1 is regulated by heat shock factor 1 and promotes glioma cells survival under heat shock stress. J Biol Chem. 2013;288:1634–42. 23192351 10.1074/jbc.M112.379362 PMC3548473

[14] Jin X, Moskophidis D, Mivechi NF. Heat shock transcription factor 1 is a key determinant of HCC development by regulating hepatic steatosis and metabolic syndrome. Cell Metab. 2011;14:91–103. 21723507 10.1016/j.cmet.2011.03.025 PMC3214631

[15] Chuma M, Sakamoto N, Nakai A, Hige S, Nakanishi M, Natsuizaka M, et al. Heat shock factor 1 accelerates hepatocellular carcinoma development by activating nuclear factor-κB/mitogen-activated protein kinase. Carcinogenesis. 2014;35:272–81. 24130164 10.1093/carcin/bgt343

[16] Li S, Ma W, Fei T, Lou Q, Zhang Y, Cui X, et al. Upregulation of heat shock factor 1 transcription activity is associated with hepatocellular carcinoma progression. Mol Med Rep. 2014;10:2313–21. 25199534 10.3892/mmr.2014.2547 PMC4214332

[17] Kawashita Y, Morine Y, Saito Y, Takasu C, Ikemoto T, Iwahashi S, et al. Role of heat shock factor 1 expression in the microenvironment of intrahepatic cholangiocarcinomas. J Gastroenterol Hepatol. 2018;33:1407–12. 29278438 10.1111/jgh.14078

[18] Dudeja V, Chugh RK, Sangwan V, Skube SJ, Mujumdar NR, Antonoff MB, et al. Prosurvival role of heat shock factor 1 in the pathogenesis of pancreatobiliary tumors. Am J Physiol Gastrointest Liver Physiol. 2011;300:G948–55. 21330448 10.1152/ajpgi.00346.2010 PMC3307520

[19] Cigliano A, Wang C, Pilo MG, Szydlowska M, Brozzetti S, Latte G, et al. Inhibition of HSF1 suppresses the growth of hepatocarcinoma cell lines in vitro and AKT-driven hepatocarcinogenesis in mice. Oncotarget. 2017;8:54149–59. 28903330 10.18632/oncotarget.16927 PMC5589569

[20] Cigliano A, Pilo MG, Li L, Latte G, Szydlowska M, Simile MM, et al. Deregulated c-Myc requires a functional HSF1 for experimental and human hepatocarcinogenesis. Oncotarget. 2017;8:90638–50. 29207593 10.18632/oncotarget.21469 PMC5710874

[21] Wang Y, Theriault JR, He H, Gong J, Calderwood SK. Expression of a dominant negative heat shock factor-1 construct inhibits aneuploidy in prostate carcinoma cells. J Biol Chem. 2004;279:32651–9. 15152009 10.1074/jbc.M401475200

[22] Fan B, Malato Y, Calvisi DF, Naqvi S, Razumilava N, Ribback S, et al. Cholangiocarcinomas can originate from hepatocytes in mice. J Clin Invest. 2012;122:2911–5. 22797301 10.1172/JCI63212 PMC3408746

[23] Zhang S, Song X, Cao D, Xu Z, Fan B, Che L, et al. Pan-mTOR inhibitor MLN0128 is effective against intrahepatic cholangiocarcinoma in mice. J Hepatol. 2017;67(6):1194–203. 28733220 10.1016/j.jhep.2017.07.006 PMC5696057

[24] Cigliano A, Zhang S, Ribback S, Steinmann S, Sini M, Ament CE, et al. The Hippo pathway effector TAZ induces intrahepatic cholangiocarcinoma in mice and is ubiquitously activated in the human disease. J Exp Clin Cancer Res. 2022;41:192. 35655220 10.1186/s13046-022-02394-2 PMC9164528

[25] Chen X, Calvisi DF. Hydrodynamic transfection for generation of novel mouse models for liver cancer research. Am J Pathol. 2014;184:912–23. 24480331 10.1016/j.ajpath.2013.12.002 PMC3969989

[26] Calvisi DF, Wang C, Ho C, Ladu S, Lee SA, Mattu S, et al. Increased lipogenesis, induced by AKT-mTORC1-RPS6 signaling, promotes development of human hepatocellular carcinoma. Gastroenterology. 2011;140:1071–83. 21147110 10.1053/j.gastro.2010.12.006 PMC3057329

[27] Scherz-Shouval R, Santagata S, Mendillo ML, Sholl LM, Ben-Aharon I, Beck AH, et al. The reprogramming of tumor stroma by HSF1 is a potent enabler of malignancy. Cell. 2014;158:564–78. 25083868 10.1016/j.cell.2014.05.045 PMC4249939

[28] Grunberg N, Levi-Galibov O, Scherz-Shouval R. The role of HSF1 and the chaperone network in the tumor microenvironment. Adv Exp Med Biol. 2020;1243:101–11. 32297214 10.1007/978-3-030-40204-4_7

[29] Grunberg N, Pevsner-Fischer M, Goshen-Lago T, Diment J, Stein Y, Lavon H, et al. Cancer-associated fibroblasts promote aggressive gastric cancer phenotypes via heat shock factor 1-mediated secretion of extracellular vesicles. Cancer Res. 2021;81:1639–53. 33547159 10.1158/0008-5472.CAN-20-2756 PMC8337092

[30] Shi Y, Sun L, Zhang R, Hu Y, Wu Y, Dong X, et al. Thrombospondin 4/integrin alpha2/HSF1 axis promotes proliferation and cancer stem-like traits of gallbladder cancer by enhancing reciprocal crosstalk between cancer-associated fibroblasts and tumor cells. J Exp Clin Cancer Res. 2021;40(1):14. 33407730 10.1186/s13046-020-01812-7 PMC7789630

[31] Mertens JC, Fingas CD, Christensen JD, Smoot RL, Bronk SF, Werneburg NW, et al. Therapeutic effects of deleting cancer-associated fibroblasts in cholangiocarcinoma. Cancer Res. 2013;73(2):897–907. 23221385 10.1158/0008-5472.CAN-12-2130 PMC3549008

[32] Tanaka N, Okada H, Yamaguchi K, Seki M, Matsubara D, Gotoh N, et al. Mint3-depletion-induced energy stress sensitizes triple-negative breast cancer to chemotherapy via HSF1 inactivation. Cell Death Dis. 2023;14:815. 38081808 10.1038/s41419-023-06352-4 PMC10713533

[33] Liao Y, Xue Y, Zhang L, Feng X, Liu W, Zhang G. Higher heat shock factor 1 expression in tumor stroma predicts poor prognosis in esophageal squamous cell carcinoma patients. J Transl Med. 2015;13:338. 26511079 10.1186/s12967-015-0703-x PMC4625739

[34] Nikotina AD, Vladimirova SA, Kokoreva NE, Nevdakha VA, Lazarev VF, Kuznetcova LS, et al. Novel mechanism of drug resistance triggered by tumor-associated macrophages through Heat Shock Factor-1 activation. Cancer Immunol Immunother. 2024;73(2):25. 38280079 10.1007/s00262-023-03612-2 PMC10821977

[35] Liu HT, Huang DA, Li MM, Liu HD, Guo K. HSF1: a mediator in metabolic alteration of hepatocellular carcinoma cells in crosstalking with tumor-associated macrophages. Am J Transl Res. 2019;11:5054–64. 31497221 PMC6731433

[36] Huang M, Dong W, Xie R, Wu J, Su Q, Li W, et al. HSF1 facilitates the multistep process of lymphatic metastasis in bladder cancer via a novel PRMT5-WDR5-dependent transcriptional program. Cancer Commun. 2022;42:447–70. 10.1002/cac2.12284 PMC9118058 35434944

[37] Hahn JS, Hu Z, Thiele DJ, Iyer VR. Genome-wide analysis of the biology of stress responses through heat shock transcription factor. Mol Cell Biol. 2004;24:5249–56. 15169889 10.1128/MCB.24.12.5249-5256.2004 PMC419887

