# Supplementary material for: HSF1 is a prognostic determinant and therapeutic target in intrahepatic cholangiocarcinoma
Source: J Exp Clin Cancer Res. 2024 Sep 6;43:253. doi: 10.1186/s13046-024-03177-7 (PMC11378393; doi:10.1186/s13046-024-03177-7)
Supplement: Supplementary file 1 — Supplementary Material 1. [file 13046_2024_3177_MOESM1_ESM.pptx]

## Slide 1
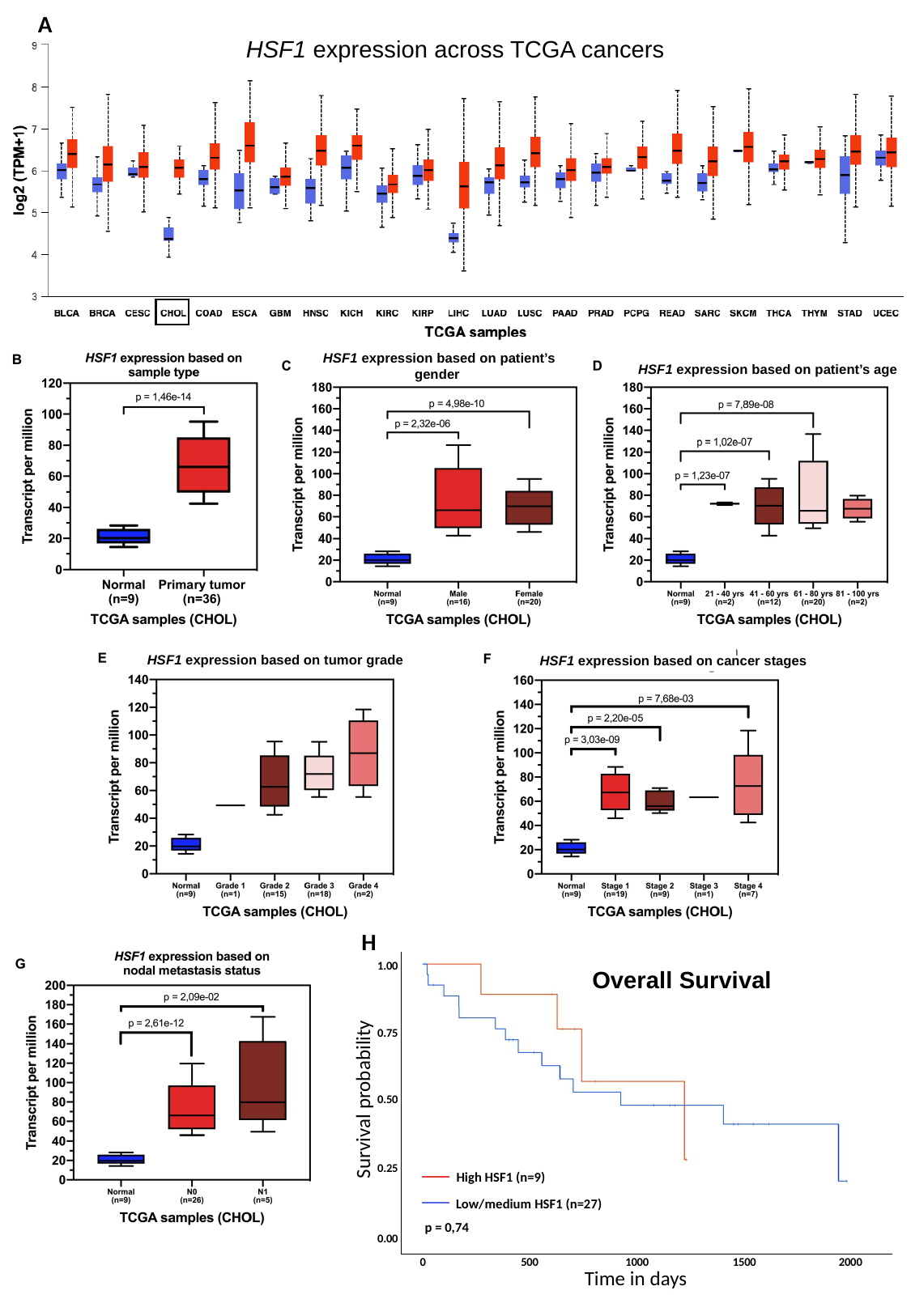

A
HSF1 expression across TCGA cancers
HSF1 expression based on patient’s gender
HSF1 expression based on patient’s age
HSF1 expression based on tumor grade
HSF1 expression based on cancer stages
H
1.00
Overall Survival
0.75
Survival probability
0.50
0.25
High HSF1 (n=9)
Low/medium HSF1 (n=27)
p = 0,74
0.00
0
500
1000
1500
2000
Time in days

## Slide 2
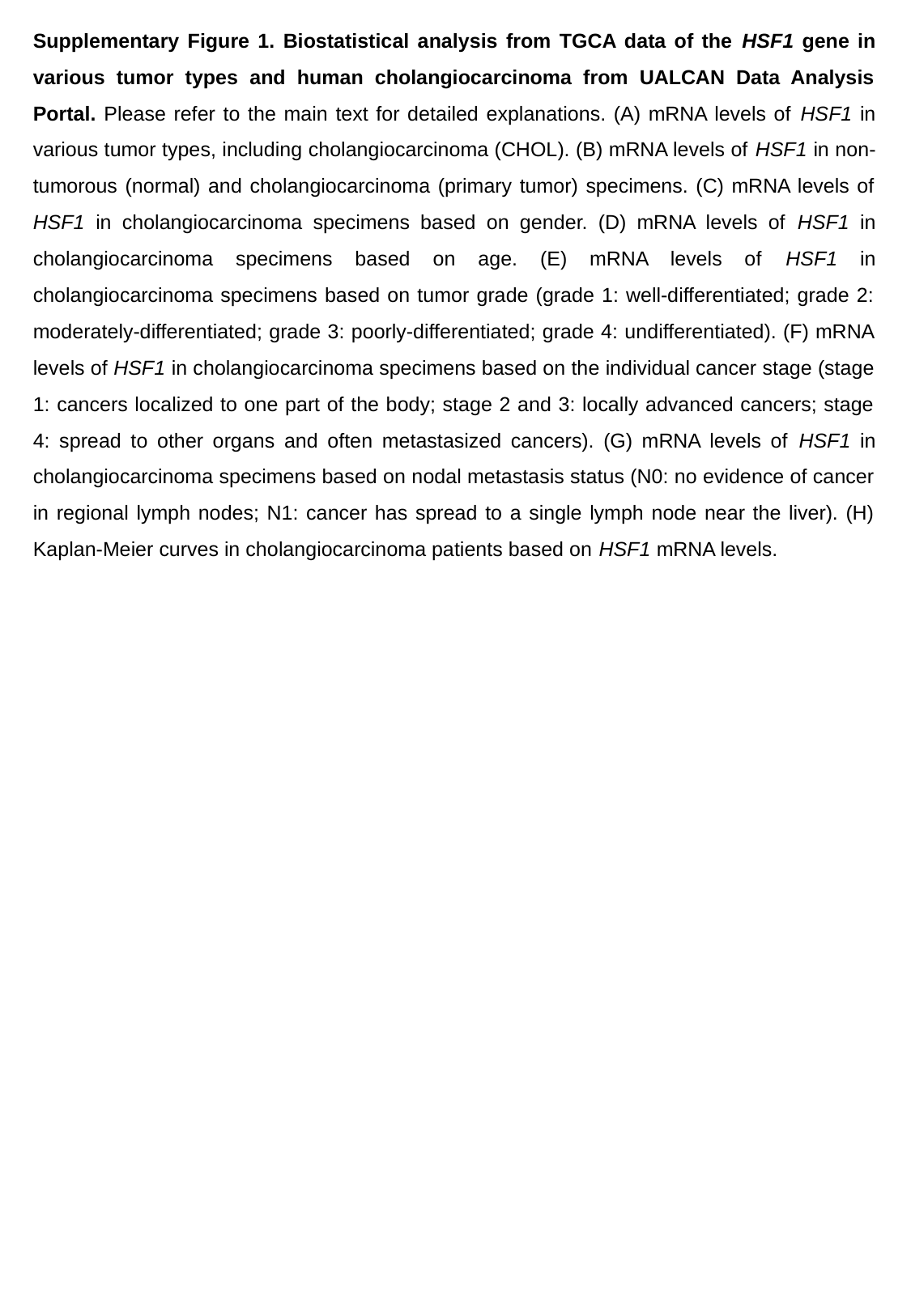

Supplementary Figure 1. Biostatistical analysis from TGCA data of the HSF1 gene in various tumor types and human cholangiocarcinoma from UALCAN Data Analysis Portal. Please refer to the main text for detailed explanations. (A) mRNA levels of HSF1 in various tumor types, including cholangiocarcinoma (CHOL). (B) mRNA levels of HSF1 in non-tumorous (normal) and cholangiocarcinoma (primary tumor) specimens. (C) mRNA levels of HSF1 in cholangiocarcinoma specimens based on gender. (D) mRNA levels of HSF1 in cholangiocarcinoma specimens based on age. (E) mRNA levels of HSF1 in cholangiocarcinoma specimens based on tumor grade (grade 1: well-differentiated; grade 2: moderately-differentiated; grade 3: poorly-differentiated; grade 4: undifferentiated). (F) mRNA levels of HSF1 in cholangiocarcinoma specimens based on the individual cancer stage (stage 1: cancers localized to one part of the body; stage 2 and 3: locally advanced cancers; stage 4: spread to other organs and often metastasized cancers). (G) mRNA levels of HSF1 in cholangiocarcinoma specimens based on nodal metastasis status (N0: no evidence of cancer in regional lymph nodes; N1: cancer has spread to a single lymph node near the liver). (H) Kaplan-Meier curves in cholangiocarcinoma patients based on HSF1 mRNA levels.
