# Supplementary material for: HSF1 is a prognostic determinant and therapeutic target in intrahepatic cholangiocarcinoma
Source: J Exp Clin Cancer Res. 2024 Sep 6;43:253. doi: 10.1186/s13046-024-03177-7 (PMC11378393; doi:10.1186/s13046-024-03177-7)
Supplement: Supplementary file 2 — Supplementary Material 2. [file 13046_2024_3177_MOESM2_ESM.pptx]

## Slide 1
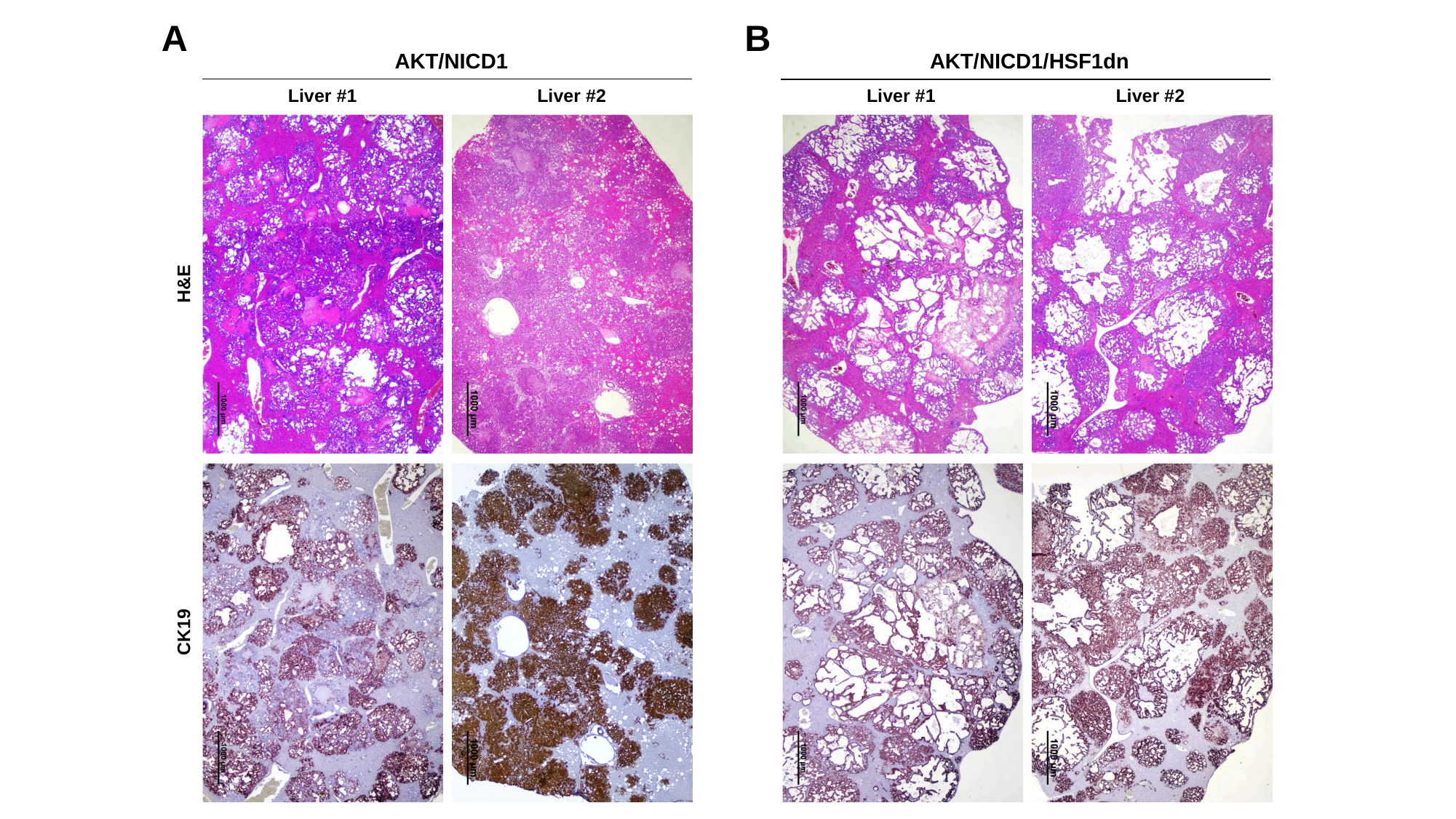

A
B
AKT/NICD1
AKT/NICD1/HSF1dn
Liver #1
Liver #2
Liver #1
Liver #2
H&E
CK19

## Slide 2
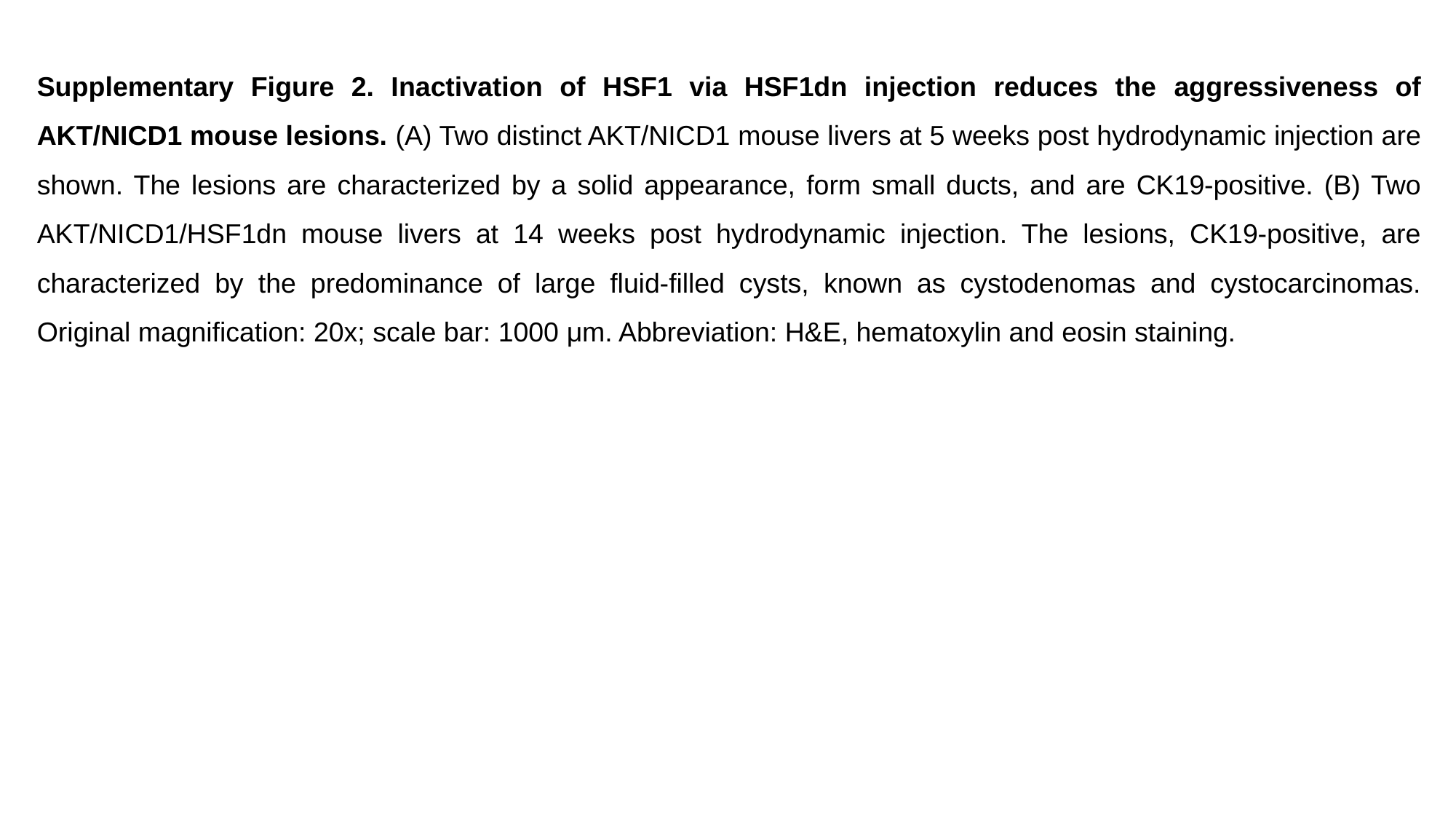

Supplementary Figure 2. Inactivation of HSF1 via HSF1dn injection reduces the aggressiveness of AKT/NICD1 mouse lesions. (A) Two distinct AKT/NICD1 mouse livers at 5 weeks post hydrodynamic injection are shown. The lesions are characterized by a solid appearance, form small ducts, and are CK19-positive. (B) Two AKT/NICD1/HSF1dn mouse livers at 14 weeks post hydrodynamic injection. The lesions, CK19-positive, are characterized by the predominance of large fluid-filled cysts, known as cystodenomas and cystocarcinomas. Original magnification: 20x; scale bar: 1000 μm. Abbreviation: H&E, hematoxylin and eosin staining.
