# Supplementary material for: HSF1 is a prognostic determinant and therapeutic target in intrahepatic cholangiocarcinoma
Source: J Exp Clin Cancer Res. 2024 Sep 6;43:253. doi: 10.1186/s13046-024-03177-7 (PMC11378393; doi:10.1186/s13046-024-03177-7)
Supplement: Supplementary file 3 — Supplementary Material 3. [file 13046_2024_3177_MOESM3_ESM.pptx]

## Slide 1
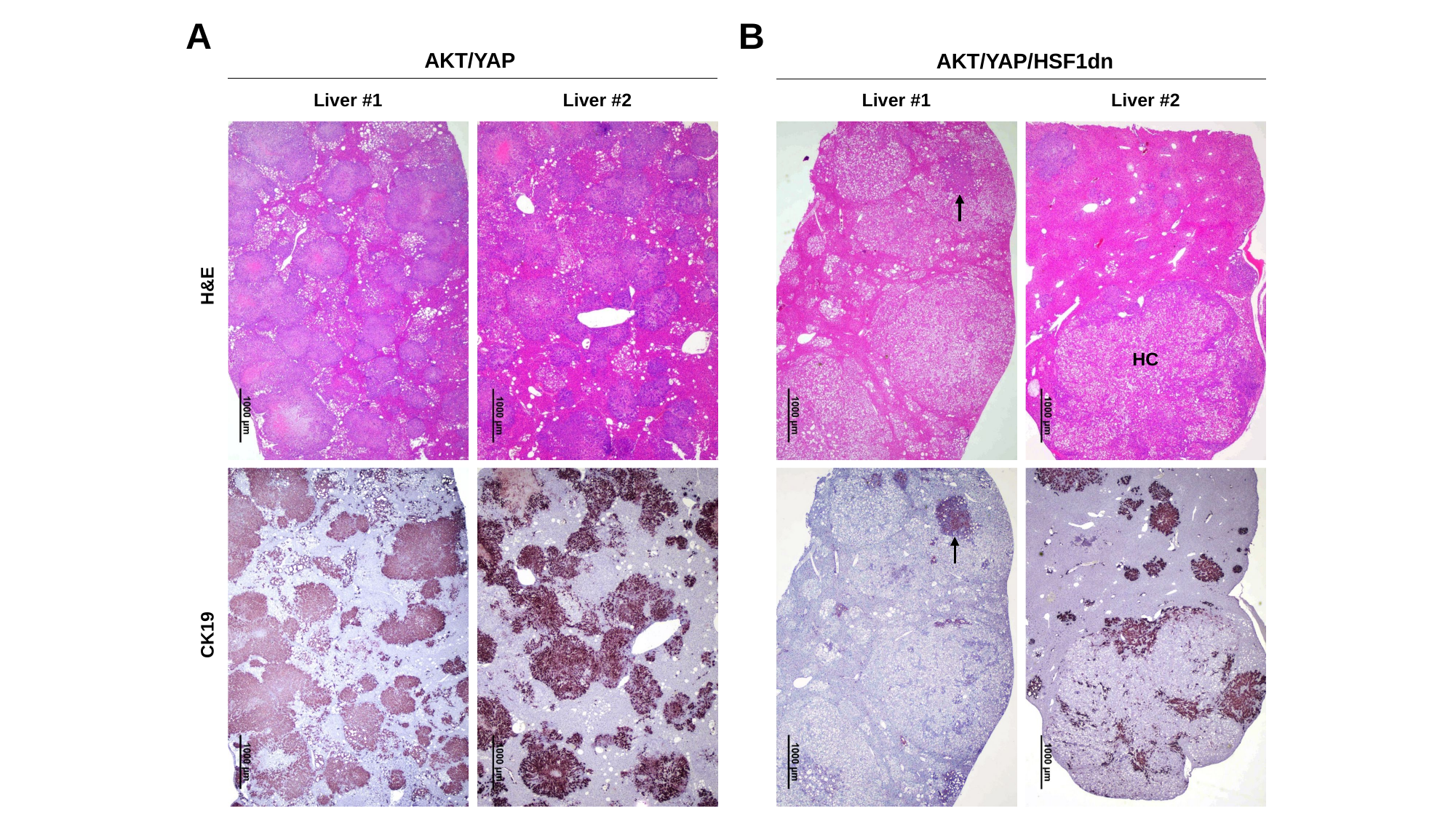

A
B
AKT/YAP
AKT/YAP/HSF1dn
Liver #1
Liver #2
Liver #1
Liver #2
H&E
HC
CK19

## Slide 2
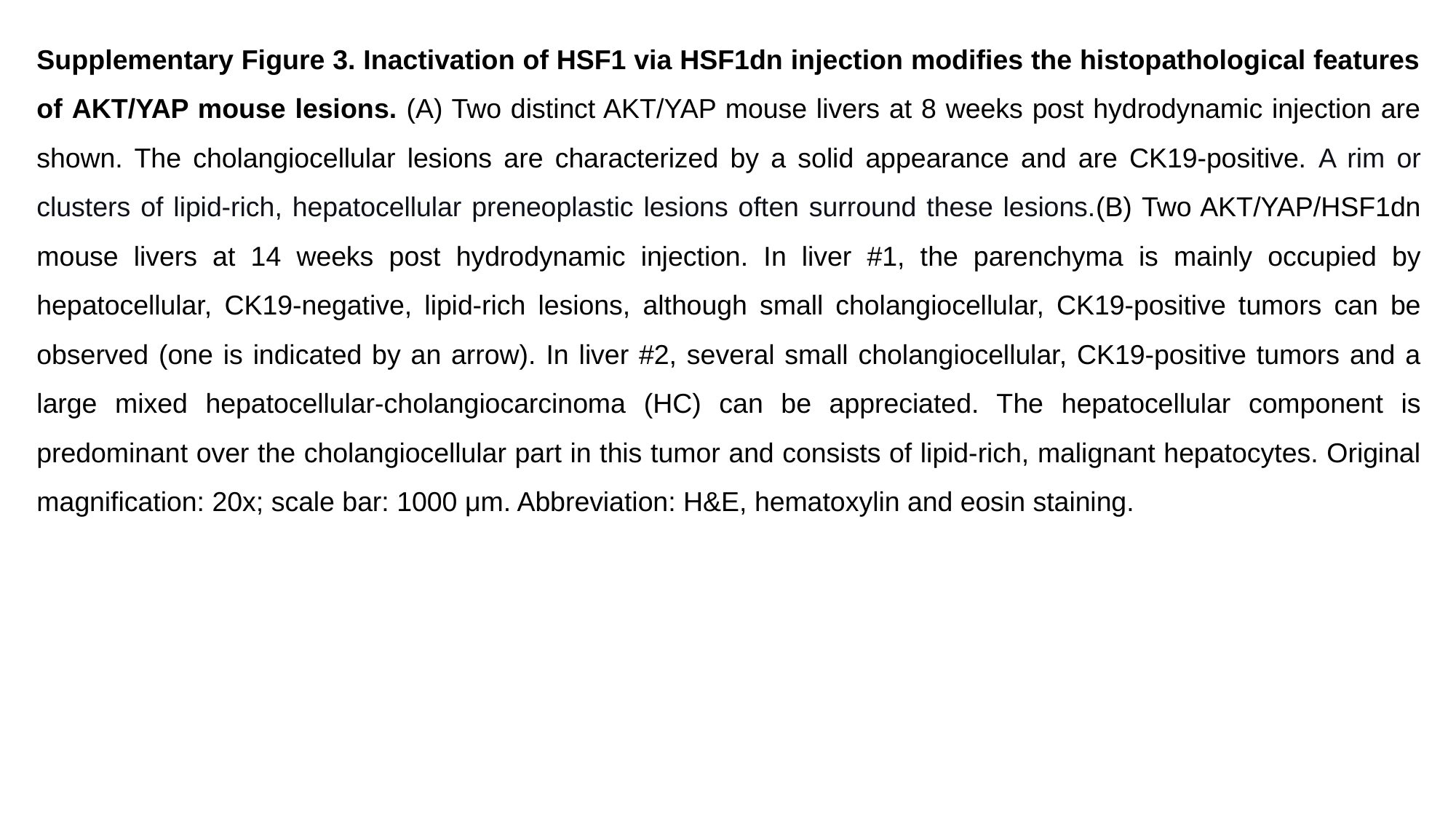

Supplementary Figure 3. Inactivation of HSF1 via HSF1dn injection modifies the histopathological features of AKT/YAP mouse lesions. (A) Two distinct AKT/YAP mouse livers at 8 weeks post hydrodynamic injection are shown. The cholangiocellular lesions are characterized by a solid appearance and are CK19-positive. A rim or clusters of lipid-rich, hepatocellular preneoplastic lesions often surround these lesions.(B) Two AKT/YAP/HSF1dn mouse livers at 14 weeks post hydrodynamic injection. In liver #1, the parenchyma is mainly occupied by hepatocellular, CK19-negative, lipid-rich lesions, although small cholangiocellular, CK19-positive tumors can be observed (one is indicated by an arrow). In liver #2, several small cholangiocellular, CK19-positive tumors and a large mixed hepatocellular-cholangiocarcinoma (HC) can be appreciated. The hepatocellular component is predominant over the cholangiocellular part in this tumor and consists of lipid-rich, malignant hepatocytes. Original magnification: 20x; scale bar: 1000 μm. Abbreviation: H&E, hematoxylin and eosin staining.
