# Supplementary material for: HSF1 is a prognostic determinant and therapeutic target in intrahepatic cholangiocarcinoma
Source: J Exp Clin Cancer Res. 2024 Sep 6;43:253. doi: 10.1186/s13046-024-03177-7 (PMC11378393; doi:10.1186/s13046-024-03177-7)
Supplement: Supplementary file 4 — Supplementary Material 4. [file 13046_2024_3177_MOESM4_ESM.pptx]

## Slide 1
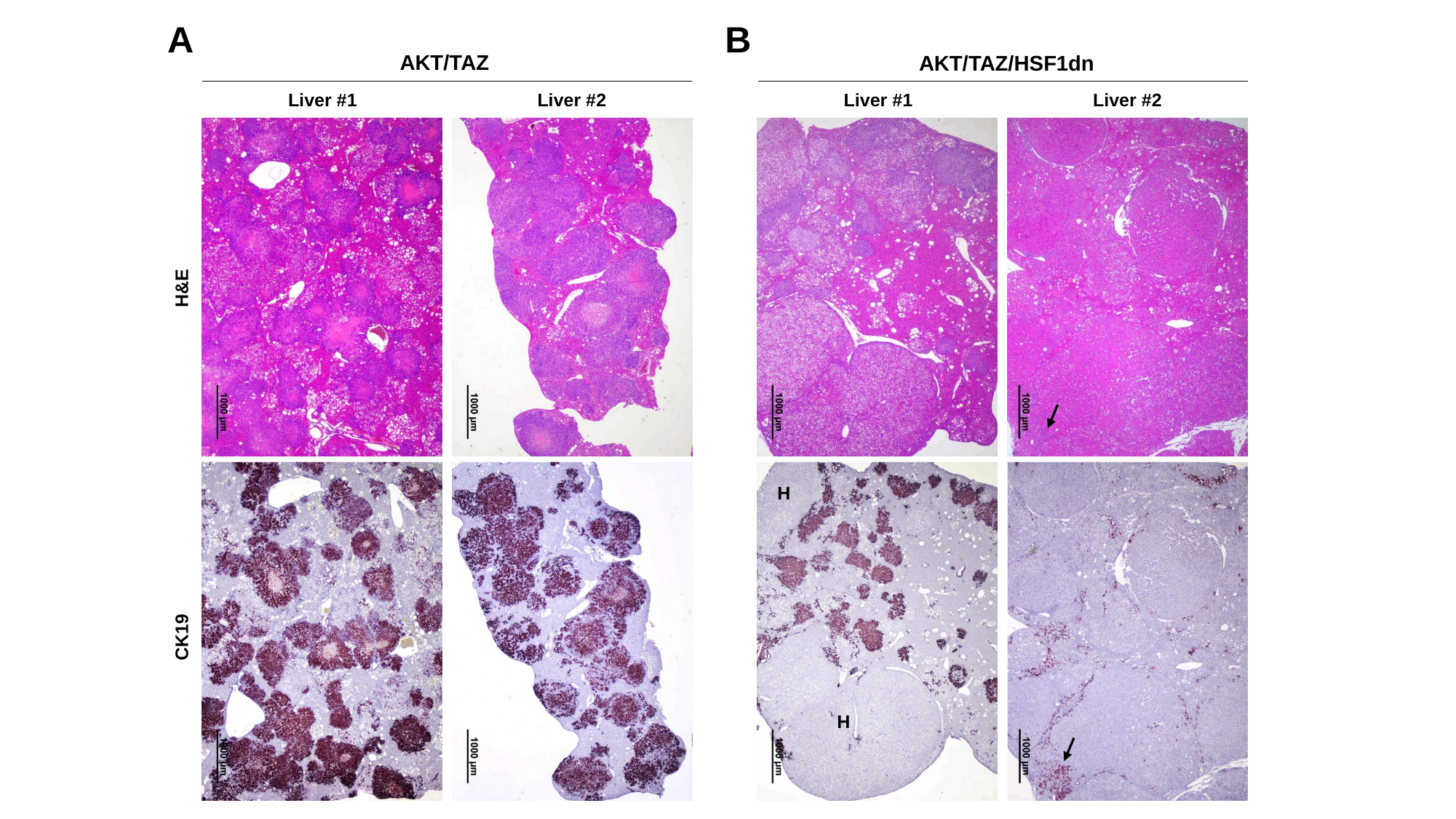

A
B
AKT/TAZ
AKT/TAZ/HSF1dn
Liver #1
Liver #2
Liver #1
Liver #2
H&E
H
CK19
H

## Slide 2
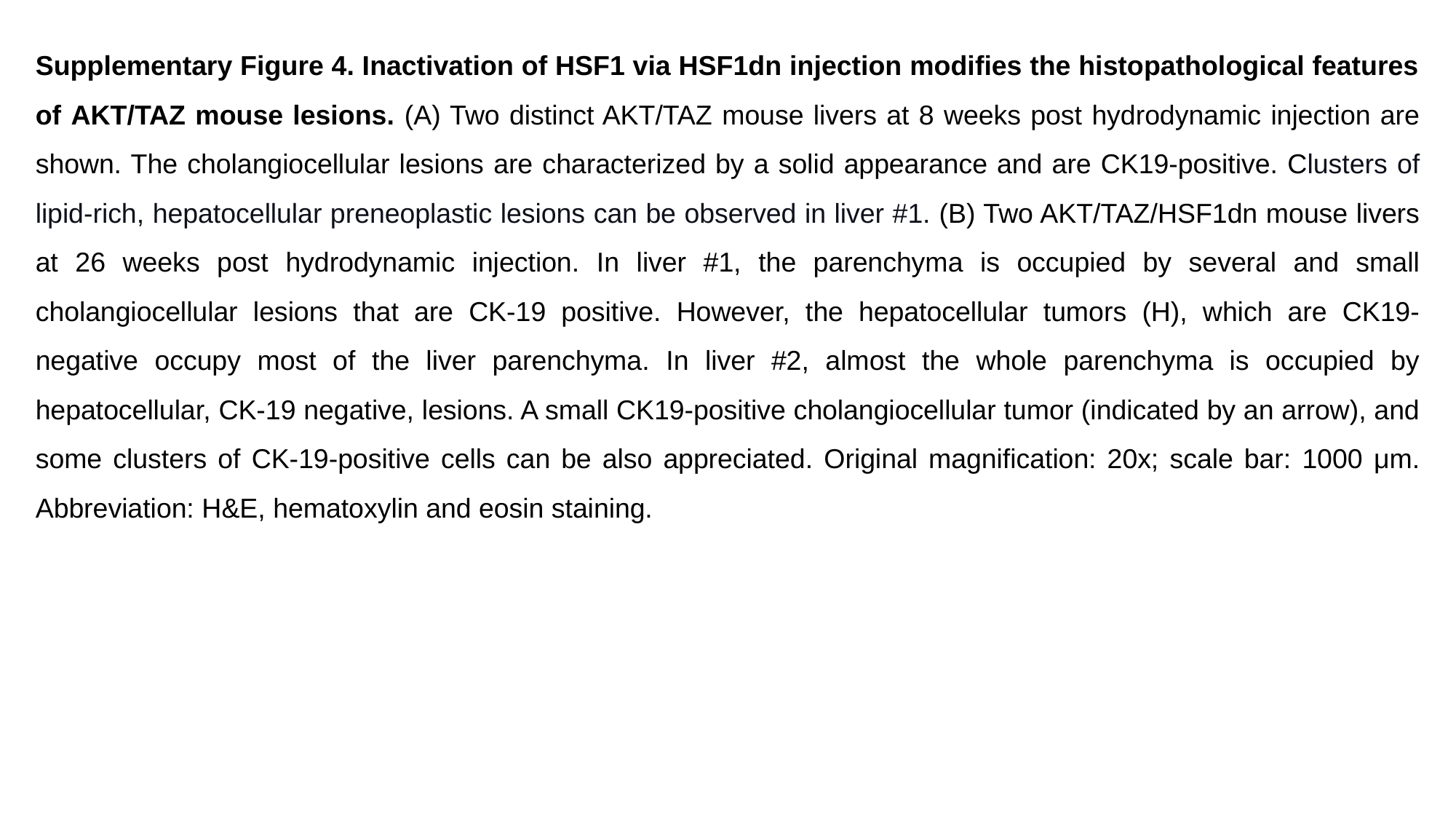

Supplementary Figure 4. Inactivation of HSF1 via HSF1dn injection modifies the histopathological features of AKT/TAZ mouse lesions. (A) Two distinct AKT/TAZ mouse livers at 8 weeks post hydrodynamic injection are shown. The cholangiocellular lesions are characterized by a solid appearance and are CK19-positive. Clusters of lipid-rich, hepatocellular preneoplastic lesions can be observed in liver #1. (B) Two AKT/TAZ/HSF1dn mouse livers at 26 weeks post hydrodynamic injection. In liver #1, the parenchyma is occupied by several and small cholangiocellular lesions that are CK-19 positive. However, the hepatocellular tumors (H), which are CK19-negative occupy most of the liver parenchyma. In liver #2, almost the whole parenchyma is occupied by hepatocellular, CK-19 negative, lesions. A small CK19-positive cholangiocellular tumor (indicated by an arrow), and some clusters of CK-19-positive cells can be also appreciated. Original magnification: 20x; scale bar: 1000 μm. Abbreviation: H&E, hematoxylin and eosin staining.
