# Supplementary material for: HSF1 is a prognostic determinant and therapeutic target in intrahepatic cholangiocarcinoma
Source: J Exp Clin Cancer Res. 2024 Sep 6;43:253. doi: 10.1186/s13046-024-03177-7 (PMC11378393; doi:10.1186/s13046-024-03177-7)
Supplement: Supplementary file 5 — Supplementary Material 5. [file 13046_2024_3177_MOESM5_ESM.pptx]

## Slide 1
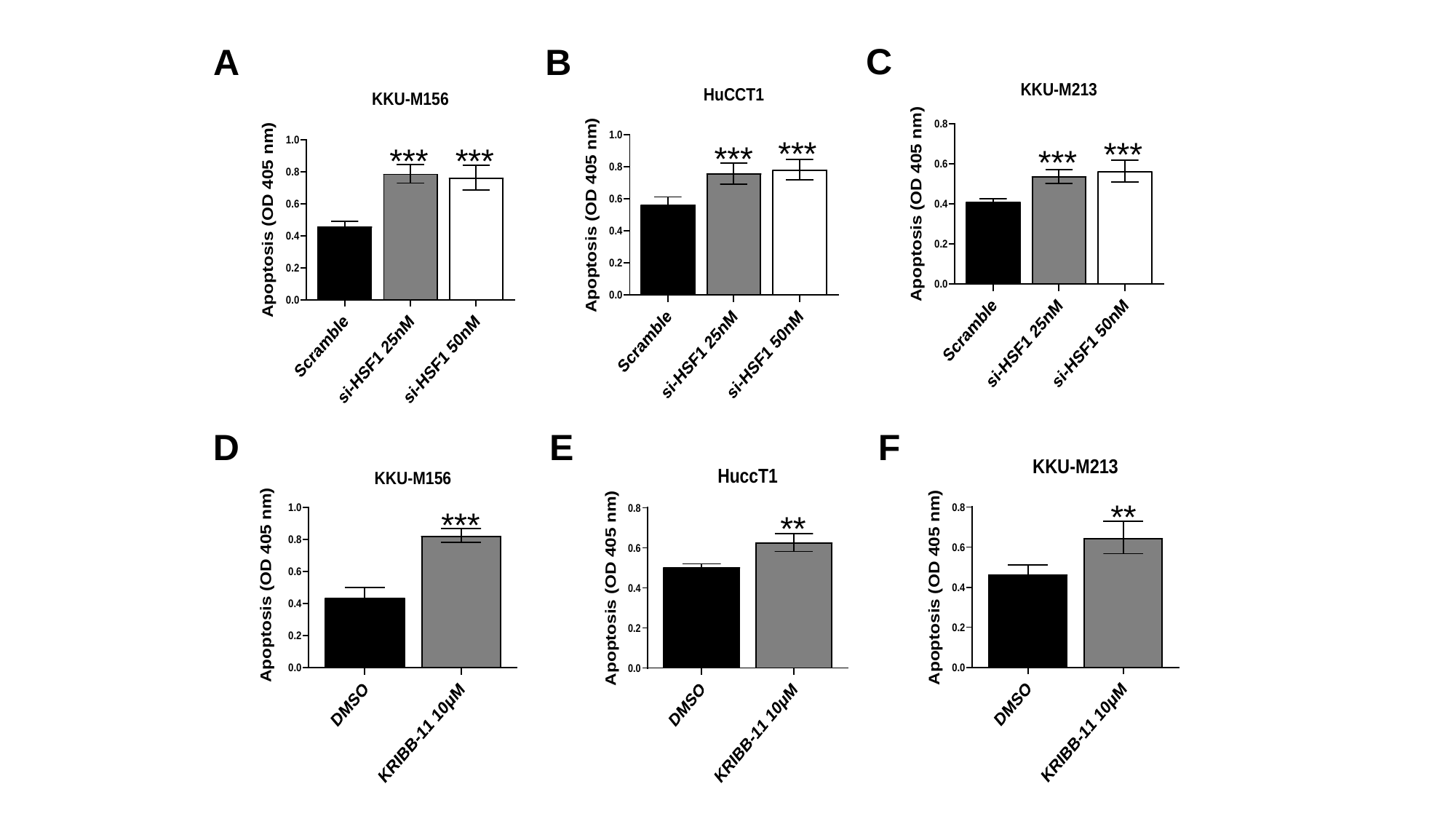

C
B
A
***
***
***
***
***
***
E
F
D
**
***
**

## Slide 2
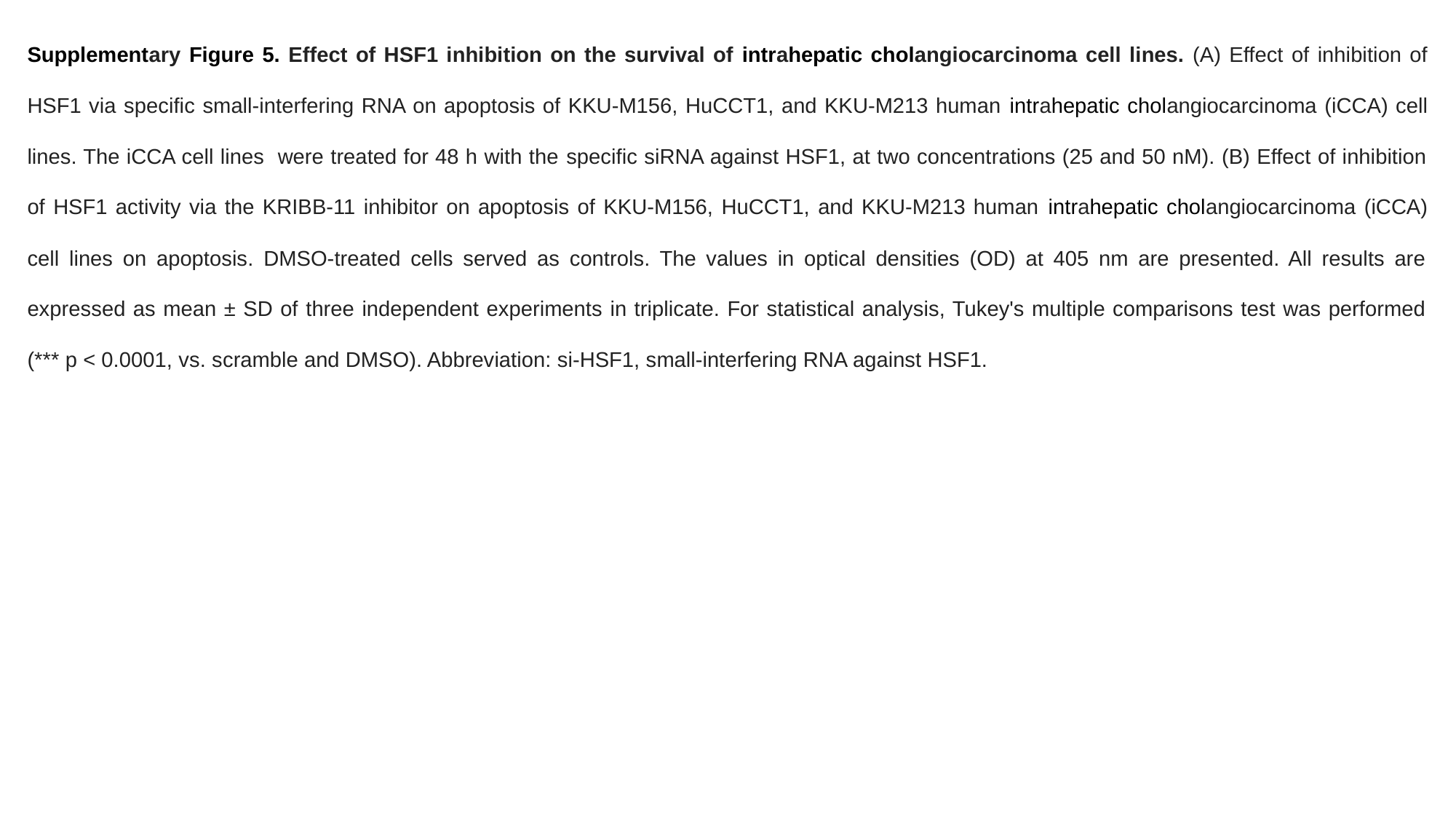

Supplementary Figure 5. Effect of HSF1 inhibition on the survival of intrahepatic cholangiocarcinoma cell lines. (A) Effect of inhibition of HSF1 via specific small-interfering RNA on apoptosis of KKU-M156, HuCCT1, and KKU-M213 human intrahepatic cholangiocarcinoma (iCCA) cell lines. The iCCA cell lines were treated for 48 h with the specific siRNA against HSF1, at two concentrations (25 and 50 nM). (B) Effect of inhibition of HSF1 activity via the KRIBB-11 inhibitor on apoptosis of KKU-M156, HuCCT1, and KKU-M213 human intrahepatic cholangiocarcinoma (iCCA) cell lines on apoptosis. DMSO-treated cells served as controls. The values in optical densities (OD) at 405 nm are presented. All results are expressed as mean ± SD of three independent experiments in triplicate. For statistical analysis, Tukey's multiple comparisons test was performed (*** p < 0.0001, vs. scramble and DMSO). Abbreviation: si-HSF1, small-interfering RNA against HSF1.
