# Supplementary material for: HSF1 is a prognostic determinant and therapeutic target in intrahepatic cholangiocarcinoma
Source: J Exp Clin Cancer Res. 2024 Sep 6;43:253. doi: 10.1186/s13046-024-03177-7 (PMC11378393; doi:10.1186/s13046-024-03177-7)
Supplement: Supplementary file 6 — Supplementary Material 6. [file 13046_2024_3177_MOESM6_ESM.pptx]

## Slide 1
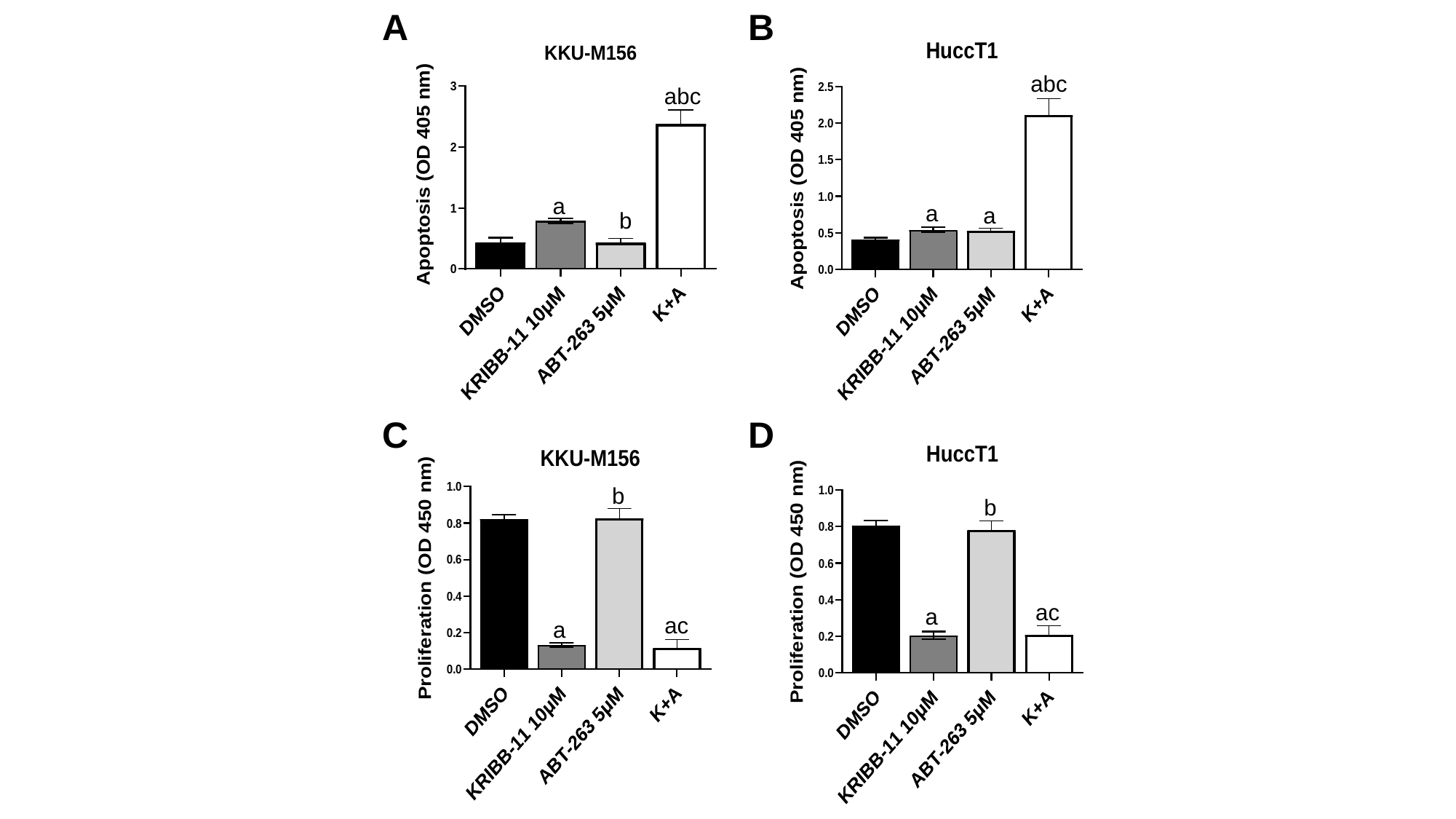

B
A
abc
abc
a
a
a
b
D
C
b
b
ac
a
ac
a

## Slide 2
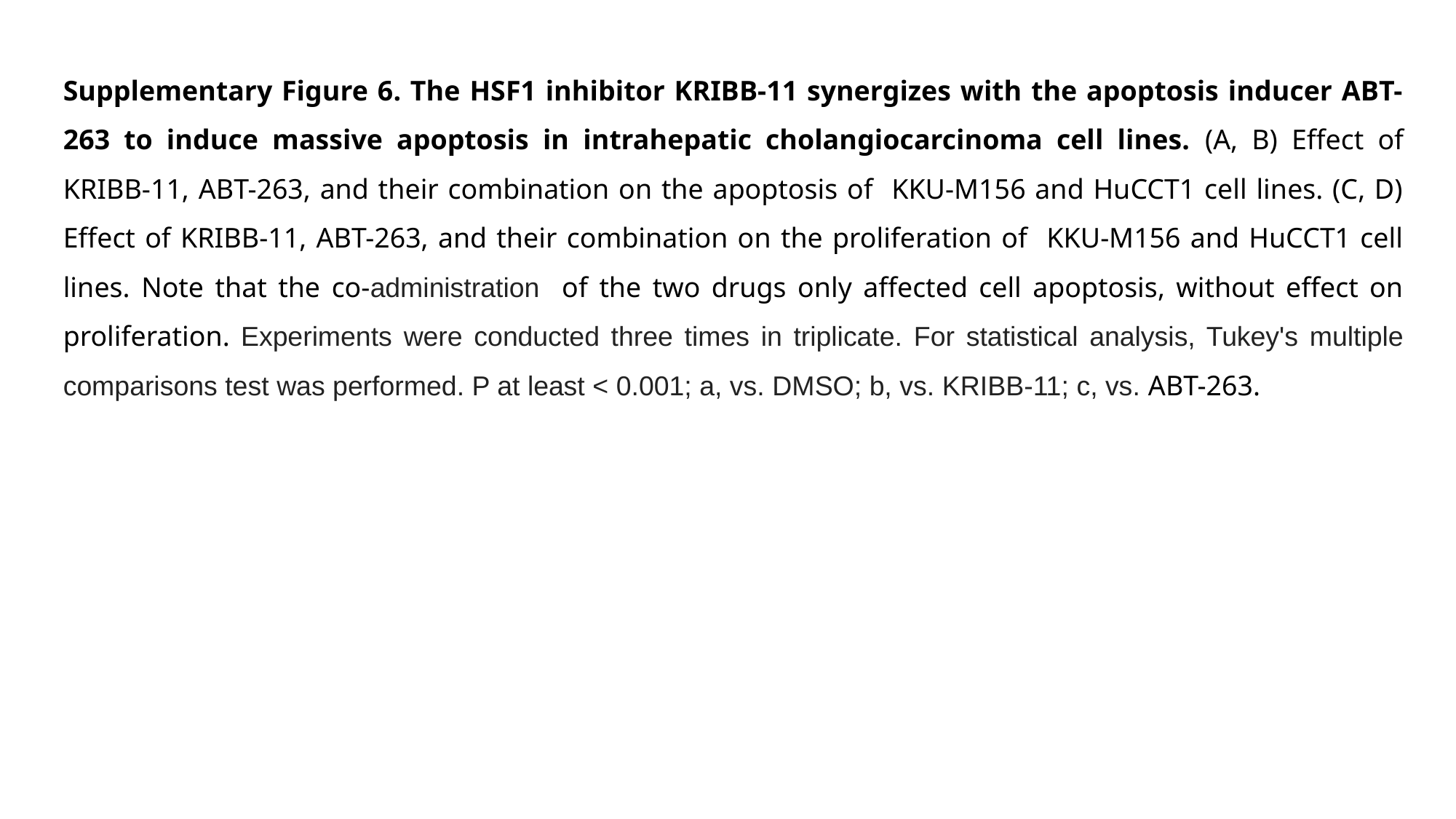

Supplementary Figure 6. The HSF1 inhibitor KRIBB-11 synergizes with the apoptosis inducer ABT-263 to induce massive apoptosis in intrahepatic cholangiocarcinoma cell lines. (A, B) Effect of KRIBB-11, ABT-263, and their combination on the apoptosis of KKU-M156 and HuCCT1 cell lines. (C, D) Effect of KRIBB-11, ABT-263, and their combination on the proliferation of KKU-M156 and HuCCT1 cell lines. Note that the co-administration of the two drugs only affected cell apoptosis, without effect on proliferation. Experiments were conducted three times in triplicate. For statistical analysis, Tukey's multiple comparisons test was performed. P at least < 0.001; a, vs. DMSO; b, vs. KRIBB-11; c, vs. ABT-263.
