# Supplementary material for: HSF1 is a prognostic determinant and therapeutic target in intrahepatic cholangiocarcinoma
Source: J Exp Clin Cancer Res. 2024 Sep 6;43:253. doi: 10.1186/s13046-024-03177-7 (PMC11378393; doi:10.1186/s13046-024-03177-7)
Supplement: Supplementary file 7 — Supplementary Material 7. [file 13046_2024_3177_MOESM7_ESM.docx]

SUPPLEMENTARY MATERIAL

The analysis included data from 72 patients with human cholangiocarcinoma (47 males and 25 females, all with survival data). The variables were analyzed using the Statistical Package for Social Science (SPSS, version 22.0, Chicago, IL, USA).

Descriptive statistics

| **Sex** | **Number (%)** | **Mean survival (SD)** |
| --- | --- | --- |
| Male | 47 (65.3) | 32.19 (15.44) |
| Female | 25 (34.7) | 29.88 (13.31) |
| Total | 72 | 31.39 (15.33) |

Mean survival is 31.39 months (SD 15.33)

(differences between males and females are not statistically significant, p=0.546)

**HSF1 in human cholangiocarcinoma**

Low and high *HSF1 mRNA* values were recoded into a binary variable (0/1) using the median values (HSF1 mRNA = 0.29900) as the cut-off. The whole dataset was then divided into 36 subjects with values of HSF1 below the median, and 36 subjects above the median. Statistical comparison between the two groups was performed using the **log-rank test**:

| **Marker** | **Number of subjects (%)** | **Mean survival in months (SD)** | **Log-rank test** |
| --- | --- | --- | --- |
| HSF1 mRNA < 0.29900 | 36 (50.0) | 41.92 (12.64) | ‒ |
| HSF1 mRNA ≥ 0.29900 | 36 (50.0) | 20.86 (9.43) | **<0.0001** |
| Total | 72 | 31.39 (15.33) |  |

Conclusion 1: *patients with HSF1 mRNA values above the median 0.29900 survive, on average, significantly shorter than patients with HSF1 mRNA below 0.29900 (see Kaplan-Meier curve).*

**Kaplan-Meier curve**

| 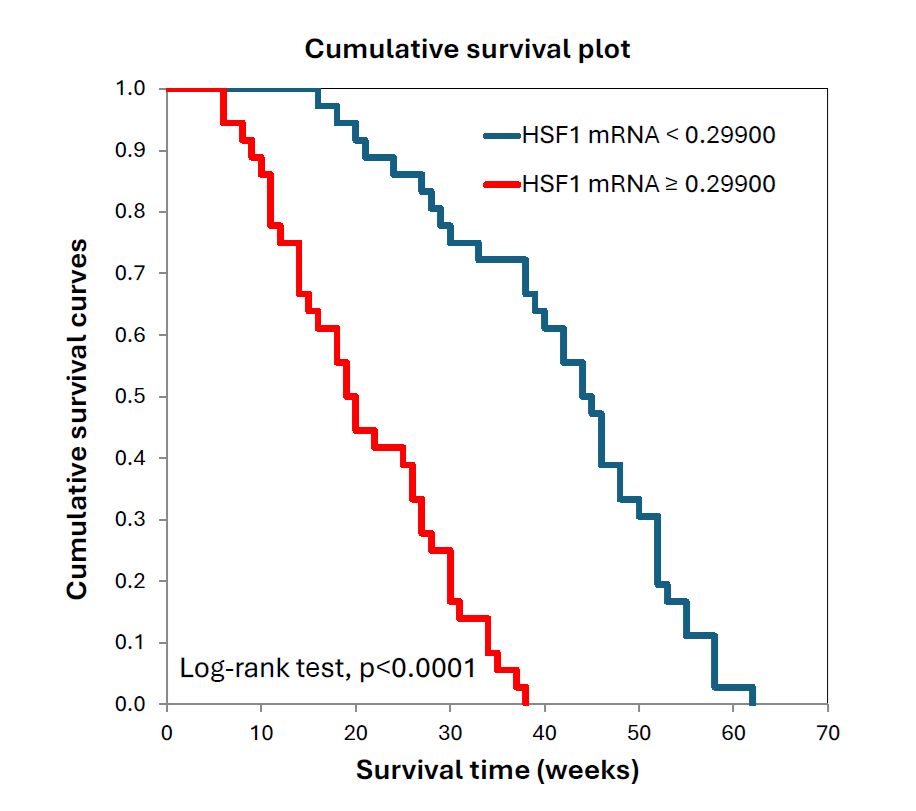 |
| --- |
| HSF1 mRNA cut-off: median |

**Survival analysis (univariate)**

| **Variable** | **Survival (months) (SD)** | **p-value** |
| --- | --- | --- |
| *Age (years)*  < 65  ≥ 65 | 31.92 (15.15)  30.86 (15.69) | 0.772 |
| *Sex*  Female  Male | 29.88 (15.31)  32.19 (15.44) | 0.546 |
| *Cirrhosis*  No  Yes | 33.63 (14.79)  25.95 (15.60) | 0.053 |
| *Etiology*  HBV  HCV  Hepatolithiasis  PSC | 26.43 (16.00)  30.70 (18.51)  27.29 (19.93)  37.33 (10.78) | 0.521^#^ |
| *Diameter*  < 5 cm  ≥ 5 cm | 30.70 (15.34)  33.32 (15.53) | 0.527 |
| *Lymph Node Metastasis*  No  Yes | 37.58 (12.65)  19.00 (12.56) | **<0.0001** |
| *Lung Metastasis*  No  Yes | 34.12 (14.66)  21.00 (13.64) | **0.003** |
| *Differentiation*  Well  Moderately  Poorly | 32.76 (16.72)  31.20 (15.27)  29.00 (12.97) | 0.706^#^ |
| *Tumor number*  Single  Multiple | 33.38 (14.74)  23.14 (15.48) | **0.024** |
| *HSF1 mRNA*  < 0.29900  ≥ 0.29900 | 41.92 (12.64)  20.86 (9.43) | **<0.0001** |

^#^One-way ANOVA

In the univariate analysis, a significant difference in survival was found only for lymph node metastasis, lung metastasis, tumor number, and HSF1 mRNA.

Differences in the dependent variable (HSF1) were evaluated with the Mann-Whitney U test for dichotomous variables, or the Kruskal-Wallis test for variables with three or more values.

| **Variable** | **No. cases** | **HSF1 mRNA** | **p-value** |
| --- | --- | --- | --- |
| *Age (years)*  < 65  ≥ 65 | 36  36 | 0.33684 ± 0.22798  0.39553 ± 0.23187 | 0.285 |
| *Sex*  Male  Female | 47  25 | 0.36409 ± 0.24470  0.37013 ± 0.20491 | 0.683 |
| *Survival*  *< 30 mo.*  *≥ 30 mo.* | 36  36 | 0.49304 ± 0.21054  0.23934 ± 0.17344 | **<0.0001** |
| *Cirrhosis*  No  Yes | 51  21 | 0.34491 ± 0.21936  0.41786 ± 0.25276 | 0.242 |
| *Etiology*  HBV  HCV  Hepatolithiasis  PSC | 14  10  7  3 | 0.35327 ± 0.22889  0.41189 ± 0.29018  0.44288 ± 0.27179  0.26640 ± 0.27059 | 0.684^#^ |
| *Diameter*  < 5 cm  ≥ 5 cm | 53  19 | 0.35239 ± 0.21484  0.40467 ± 0.27127 | 0.596 |
| *Lymph Node Metastasis*  No  Yes | 48  24 | 0.26513 ± 0.16469  0.56830 ± 0.21034 | **<0.0001** |
| *Lung Metastasis*  No  Yes | 57  15 | 0.31330 ± 0.20329  0.56717 ± 0.22091 | **<0.0001** |
| *Differentiation*  Well  Moderately  Poorly | 34  20  18 | 0.32538 ± 0.22201  0.40605 ± 0.24632  0.39898 ± 0.22697 | 0.367^#^ |
| *Tumor number*  Single  Multiple | 58  14 | 0.34276 ± 0.22351  0.46325 ± 0.24042 | 0.083 |

^#^Kuskal-Wallis test

A significant difference in HSF1 mRNA level was found only for survival, lymph node metastasis, and lung metastasis.

Correlation analysis was performed by calculating Spearman's coefficient.

| **Variable** | ***HSF1* mRNA** | **p-value** |
| --- | --- | --- |
| *Age* | 0.201 | 0.090 |
| *Sex* | 0.048 | 0.686 |
| *Survival* | –0.771 | **<0.0001** |
| *Cirrhosis* | 0.139 | 0.244 |
| *Etiology* | –0.027 | 0.822 |
| *Diameter* | 0.063 | 0.599 |
| *Lymph Node Metastasis* | 0.598 | **<0.0001** |
| *Lung Metastasis* | 0.427 | **<0.0001** |
| *Differentiation* | –0.161 | 0.176 |
| *Tumor number* | 0.206 | 0.083 |
| *Ki67* | 0.539 | **<0.0001** |

**Conclusion: *HSF1* mRNA shows a significant negative correlation with patient survival and a significant positive correlation with lymph node, lung metastases, and Ki67.**
